# Continuous *in vivo* Metabolism by NMR

**DOI:** 10.3389/fmolb.2019.00026

**Published:** 2019-04-30

**Authors:** Michael T. Judge, Yue Wu, Fariba Tayyari, Ayuna Hattori, John Glushka, Takahiro Ito, Jonathan Arnold, Arthur S. Edison

**Affiliations:** ^1^Department of Genetics, University of Georgia, Athens, GA, United States; ^2^Institute of Bioinformatics, University of Georgia, Athens, GA, United States; ^3^Department of Biochemistry and Molecular Biology, University of Georgia, Athens, GA, United States; ^4^Complex Carbohydrate Research Center, University of Georgia, Athens, GA, United States; ^5^Division of Hematological Malignancy, National Cancer Center Research Institute, Tokyo, Japan

**Keywords:** CIVM-NMR, real-time metabolomics, dynamics, flux, HR-MAS, myeloid leukemia cells, *Neurospora crassa*, BCAA

## Abstract

Dense time-series metabolomics data are essential for unraveling the underlying dynamic properties of metabolism. Here we extend high-resolution-magic angle spinning (HR-MAS) to enable continuous *in vivo* monitoring of metabolism by NMR (CIVM-NMR) and provide analysis tools for these data. First, we reproduced a result in human chronic lymphoid leukemia cells by using isotope-edited CIVM-NMR to rapidly and unambiguously demonstrate unidirectional flux in branched-chain amino acid metabolism. We then collected untargeted CIVM-NMR datasets for *Neurospora crassa*, a classic multicellular model organism, and uncovered dynamics between central carbon metabolism, amino acid metabolism, energy storage molecules, and lipid and cell wall precursors. Virtually no sample preparation was required to yield a dynamic metabolic fingerprint over hours to days at ~4-min temporal resolution with little noise. CIVM-NMR is simple and readily adapted to different types of cells and microorganisms, offering an experimental complement to kinetic models of metabolism for diverse biological systems.

## Introduction

Metabolic time-series data are invaluable for the development and validation of high-quality models that accurately describe the dynamics of metabolism (Montana et al., 2011; Link et al., 2014; Sefer et al., 2016). Information about the changing metabolic state of an organism typically requires extensive time, resources, and sample material. As such, researchers must choose between variables such as the number of replicates, the experiment duration, and the time resolution for time-series. Furthermore, traditional metabolomics experimental designs face the challenges of extraction biases (Sitnikov et al., 2016) and the confounding of biological and analytical variance (Tabatabaei Anaraki et al., 2018). While many studies employ sample preparation and extraction approaches effectively, direct or *in vivo* measurements are fundamentally simpler to obtain and interpret. Likewise, while carefully designed (Rhoades et al., 2017) and executed studies with large sample sizes yield powerful insights into the dynamics of biological systems (Sengupta et al., 2016; Krishnaiah et al., 2017; Cannon et al., 2018), continuous and repeated measurements on the same living sample are invaluable for monitoring and confirming these dynamics.

Small molecules and their fluxes have been measured *in vivo* using NMR (Bastawrous et al., 2018), and methods have recently been developed that begin to address the need for a continuous time dimension in metabolomics data. For example, long-standing flow NMR techniques allow monitoring of secretion and uptake of extracellular metabolites for organisms grown in liquid culture (Bastawrous et al., 2018). Link et al. recently achieved high temporal resolution on many metabolites by developing an automated real-time metabolomics platform that samples liquid cultures of single cells and directly injects them onto a time-of-flight mass spectrometer every 15–30 s (Link et al., 2015). The group have more recently probed the interactions between biomass synthesis and cell division in *E. coli* using this method (Sekar et al., 2018). Koczula et al. conducted *in vivo* measurements changes in media composition with 4–8 min resolution for chronic lymphoid leukemia. Sedimentation and line broadening are major factors that limit standard NMR measurements of complex samples like cells. Koczula et al. were able to mitigate sedimentation by immobilizing the single cells in agarose (Koczula et al., 2016).

Alternatively, HR-MAS enables high-resolution NMR measurements on mixed-phase samples such as tissues (Beckonert et al., 2010), or more recently, living organisms (Righi et al., 2014; Sarou-Kanian et al., 2015; Augustijn et al., 2016; Mobarhan et al., 2016; Bastawrous et al., 2018) with minimal line broadening. In this study, we extended HR-MAS to real-time continuous *in vivo* measurements of metabolism in cells. Using isotope editing, CIVM-NMR was able to reproduce and more directly observe a surprising branched-chain amino acid (BCAA) flux result reported last year in human myeloid leukemia cells (Hattori et al., 2017). We found that CIVM-NMR was not only easier but faster and more conclusive than traditional approaches for flux measurements in human cell cultures. We then applied CIVM-NMR to the multicellular filamentous fungus, *N. crassa*, in both aerobic and anaerobic environments. We observed highly reproducible dynamics in central carbon and amino acid metabolism with ~4 min resolution over 11 h. The continuous nature of these measurements facilitated metabolite annotation, and semi-automated peak tracing provided relative quantification of known and unknown compounds. We developed several new MATLAB functions and workflows, freely available through GitHub, for the analysis and visualization of these novel data. As CIVM-NMR can be applied widely to cells, tissues, and small multicellular organisms, it enables new opportunities in fields such as developmental and chronobiology for monitoring high-resolution metabolic time-series data. Importantly, it will enable more robust and experimentally-based kinetic metabolic models for diverse biological systems.

## Materials and Methods

### Human Leukemia Cell Culture and Preparation for HR-MAS NMR

The human BC-CML cell line K562 was obtained from ATCC, and cell line authentication testing was performed by ATCC-standardized STR analysis to verify their identity. After cell counting and washing with PBS, K562 cells were resuspended and labeled in a custom-made Iscove's modified Dulbecco's Medium (IMDM) without BCAAs supplemented with 10% dialyzed FBS, 100 IU/ml penicillin, 100 μg/ml streptomycin, and the following amino and keto acids: For ^13^C-KIV (keto-isovalerate) tracer experiments, isoleucine, leucine and valine were supplemented at 170 μM. For ^13^C-valine tracer experiments, isoleucine, leucine, and KIV were added at 170 μM. Cell suspension (54 μl) was loaded in a clean 4 mm diameter zirconia HR-MAS rotor (Bruker BioSpin), and then either [(U)-^13^C]-ketoisovalerate or [(U)-^13^C]-valine solution in D_2_O was added to a final concentration of 170 μM. The rotor was sealed with a Kel-F rotor cap (Bruker BioSpin).

### Preparation of Growth Media and Slants for *N. crassa*

Ingredients for Vogel's media (3 % glucose) (glucose, 0.167 M; biotin, 0.614 μM; arginine, 1.95 mM; Na_3_ citrate, 9.74 mM; KH_2_PO_4_, 36.7 mM; NH_4_NO_3_, 25.0 mM; MgSO_4_, 0.811 mM; CaCl_2_, 0.680 mM; ZnSO_4_, 34.8 μM; Fe (NH_4_)_2_ (SO_4_)_2_, 5.10 μM; CuSO_4_, 2.00 μM; MnSO_4_, 0.592 μM; H_3_BO_3_, 1.62 μM; Na_2_MoO_4_, 0.413 μM) were dissolved in ddH_2_O in a large glass bottle, mixed by stirring, filter-sterilized (0.22 μm Steritop threaded bottle top filter, 500 mL, Millipore EMD), then aliquoted into clean, sterile 500-mL bottles. Ingredients for Vogel's media with agar (same as above, with the addition of 1.5% agar, w/v, and using 1.5% glucose, w/v) were combined in a beaker. Agar was dissolved by heating in a microwave oven. The dissolved mixture was aliquoted to 15-mL or 5-mL glass test tubes, stoppered with cotton, and sterilized by autoclaving.

### Vogel's Media for NMR and Wash Solution

2X Vogel's media (minus glucose), DSS solution, and D_2_O were combined to make a concentrate, which was split into two aliquots. To prepare Vogel's media for NMR (1.5% glucose), filter-sterilized D-glucose solution (0.5 mg/μL) was added to the smaller aliquot to a final composition of glucose, 83 mM; DSS, 1 mM; biotin, 0.614 μM; L-arginine, 1.95 mM; Na_3_ citrate, 9.74 mM; KH_2_PO_4_, 36.7 mM; NH_4_NO_3_, 25.0 mM; MgSO_4_, 0.811 mM; CaCl_2_, 0.680 mM; ZnSO_4_, 34.8 μM; Fe (NH_4_)_2_ (SO_4_)_2_, 5.10 μM; CuSO_4_, 2.00 μM; MnSO_4_, 0.592 μM; H_3_BO_3_, 1.62 μM; Na_2_MoO_4_, 0.413 μM in 95 ddH_2_O/5 D_2_O (v/v). Wash solution was prepared by adding ddH2O in place of D-glucose solution to the larger aliquot.

### Preparation and Storage of *N. crassa* Conidial Suspension

A frozen *bd1858* (A) stock obtained by the Fungal Genetics Stock Center (McCluskey et al., 2010) was used to inoculate two growth slants (Vogel's media agar, 1.6% glucose w/v, 3 mL in 15 mL glass test tubes stoppered with sterile cotton plugs). These were incubated for 2 days at 30°C, then placed under a benchtop lamp at 25°C for 2 days to induce maturation of conidia. Conidia were collected from both tubes sequentially by suspension in 12 mL Vogel's media (no glucose) and filtration through sterile cotton. Concentration of the resulting conidial suspension was found to be 6.47 × 10^7^ cells/mL using a Nexus Cellometer Auto 2000 (Nexelcom Bioscience; Lawrence, MA, USA). The conidial suspension was kept at 4°C over the course of the experiments (4 weeks).

### Growth of *N. crassa* Mycelia

Vogel's media (50 mL, 3% glucose w/v) in a 250-mL Erlenmeyer flask was inoculated under aseptic conditions with conidial suspension to a total concentration of 2.7 × 10^4^ cells/mL (21 μL conidial suspension), and covered with aluminum foil. Liquid cultures were grown with orbital shaking (~237 rpm) at room temperature (~25°C) under constant cool white light (7 μmol L^−1^ s^−1^ m^−2^) for 32 h. At that point mycelia consistently formed a single, cohesive mass. Mycelia for ^13^C glucose experiments were allowed to grow for 48–52 h. The entire culture was transferred to a 50 mL conical tube (Sarstedt; Newton, NC, USA) for transport to the NMR facility (15–30 min).

### Preparation of *N. crassa* Mycelia

Under aseptic conditions, a section of mycelium from the edge of the main mycelial mat was cut off using a sterile tube cap and trimmed to fit the volume of approximately 126 μL using a pre-marked microcentrifuge tube. Mycelia were handled from this point using clean, sterile tweezers (cleaned with 70% EtOH on a lint-free single-ply lab tissue (Kimwipe) and dried in an aseptic environment). The section of mycelium was then patted dry on autoclaved filter paper (Whatman Filter Paper #3; GE Healthcare, USA) atop a layer of folded Kimwipes, and was washed by placing in a sterile microcentrifuge tube containing 1 mL wash solution and vortexing briefly (~10 s) until the mycelium had fully absorbed the media. Washing was repeated with fresh wash solution for a total of 4 washes. The mycelium was reduced to ~63 μL (0.9 × volume of rotor + plug), measured in a second microcentrifuge tube pre-marked to that volume. The mycelium was pat-dried in a sandwich of sterile filter paper folded into Kimwipes, pressing firmly three times (until no liquid spots were visible on the filter paper). The dried mycelium was then weighed in a separate microcentrifuge tube. The dry mycelium was 9.04–10.13 mg in our experiments (μ = 9.62 mg; SD = 0.32 mg). We observed a reduction in mass of ~30% as conidia, loose filaments, and other debris are removed along with waste products and glucose during wash steps. In our hands, the prep process took between 4 and 13 min, during which time the organism was immersed in a low-glucose environment.

### Loading *N. crassa* Mycelia Into the Rotor

The dried, weighed mycelium was then placed in a microcentrifuge tube containing fresh Vogel's media for NMR (500 μL, 1.5% glucose), and vortexed briefly until the mycelium had fully absorbed the media. The mycelium was then transferred to a third, pre-marked microcentrifuge tube (63 μL). By adding/removing media, the volume was adjusted to the 63 μL volume mark. Sterile tweezers were used to transfer the mycelium to a clean 4 mm diameter zirconia rotor (Bruker BioSpin) cleaned by rinsing with bleach solution, tap water, 70% ethanol, tap water, and ddH_2_O x 4). The mycelium was pushed to the bottom, taking caution not to lose liquid. The remaining liquid in the tube was added to the rotor and one tweezer prong was used to position the mycelium to remove larger air bubbles, although small bubbles occurred with no issues in the NMR. A teflon sealing plug (Bruker BioSpin) was then inserted to ~2 mm below the edge of the rotor. For the aerobic condition, a Kel-F rotor cap (Bruker BioSpin) modified with a 0.016-inch diameter hole drilled using a lathe was lined on the inside with three layers of rayon breathable microplate sealing tape (QuickSeal breathable film, Thomas Scientific, USA) to prevent spore escape. The cap was fully inserted to push the sealing plug into its final position. The cap was then removed, and the insides of the cap and plug were inspected to ensure that no liquid was lost and that an airspace existed between the plug and the sample. The rotor was then re-capped, the bottom edge marked with a permanent marker, and dropped into the bore of the magnet (cap facing up). In our hands, this process typically takes 15–30 min. For the anaerobic condition, media was added to fill all airspaces and an unmodified cap was used to prevent gas exchange. For the ^13^C labeling experiments in aerobic conditions, an airspace was left and fresh Vogel's media for NMR was prepared (minus citrate and glucose). Within 3 min before measurements, ^13^C-labeled glucose (99% labeled; Cambridge Isotope Laboratories; Tewksbury, MA, USA) was added to a final concentration of 1.5% (w/v) or 83 mM without adjusting the concentration of other media components.

### NMR Parameters

For human ML cell experiments, a hsqcetgpsisp gradient heteronuclear single quantum coherence spectroscopy (HSQC) experiment run as a 1D experiment was used with the following parameters: number of points: 7272; dummy scans; 4 at the beginning of the run; number of scans: 128/timepoint. O1 offset: 4.699 ppm; O2: 30 ppm; acquisition time 0.3999600 s; recycle delay: 1.5 s; receiver gain: auto (101); temperature: 298 K = 25°C; spinning speed: 3,100 Hz. A standard noesypr1d protocol (Bruker) was used for *N. crassa* non-labeled real-time metabolomics measurements. The following parameters applied to all samples and timepoints: data points: 42856; dummy scans: 8; number of scans: 64/timepoint; spectral width 19.8395 ppm; acquisition time 1.7999520 s; recycle delay: 1.5 s.; receiver gain: auto (101); temperature: 298 K = 25°C [calibrated using a deuterated methanol standard (Van Geet, 1970)]. The following parameters were optimized for each sample: O1 offset for water suppression: 4.695–4.697 ppm. PWL9 water suppression power: 43.87–44.42 dB (μ = 44.23 dB, SD = 0.19 dB). P1 pulse width: 12.49–13.30 μs (μ = 12.78 μs, SD = 0.29 μs). Spinning speed: 6,000 Hz. Notably, this variation in pulse width between samples manifested as a difference in temporal resolution (i.e., longer pulse widths resulted in time points slightly farther apart). The effect was measurable (on the order of minutes) over hundreds of measurements. The average experiment took 4.23 ± 0.004 min (SD).

For measurement of ^13^C in the labeled glucose experiment, a hsqcetgpsisp gradient heteronuclear single quantum coherence spectroscopy (HSQC) experiment run as a 1D experiment was used with the following parameters: number of points: 4686; dummy scans: 4; number of scans: 8/timepoint; O1 offset: 4.695 ppm; O2 offset: 75.001 ppm; spectral width 13.0208 ppm; acquisition time 0.2999040 s; recycle delay: 1.5 s; receiver gain: auto (101); temperature: 298 K = 25°C; spinning speed: 3,500 Hz. The ^13^C experiments were interleaved with noesypr1d experiments as described above, but with 4 dummy scans and 8 scans, resulting in a resolution of 2 min. All Bruker parameter files are available with the raw and processed data at http://www.metabolomicsworkbench.org/.

### Automated Data Acquisition and Post-experiment Sample Preparation

For human ML cells, spectra were collected sequentially using the multizg command in TopSpin (v4.0.1; Bruker).

For *N. crassa* samples, the noesypr1d experiment, optimized for the sample, was imported into IconNMR in TopSpin (v4.0.1; Bruker). The solvent was set to “D2O_H2O+salt.” The “iterate” command was used to queue 1024 identical, sequential noesypr1d experiments (each taking ~4.6 min) on a 600 MHz Bruker NEO equipped with a 4-mm CMP-MAS probe. Experiments generally ended after ~12 h, although some were allowed to continue as long as 37 h. By spinning *N. crassa* at 6 KHz, spinning sidebands (Maricq and Waugh, 1979) were eliminated in the spectral region of 0–10 ppm. At the end of each run, the mycelia were transferred from the rotor to a sterile microcentrifuge tube with clean, sterile tweezers. All liquid from the rotor was also transferred to the tube. This was either extracted and assessed for growth immediately or was allowed to sit on the bench for 1 day.

### Survival Assessment

Sterile tweezers were used to tear a piece of mycelium from the rotor contents; this was used to inoculate a growth slant. All growth slants were assessed for 24 h or longer post-inoculation for growth. Photographs were taken using a 16 MP digital camera on an LG G5 cell phone in Manual Mode.

### Extraction

The remaining rotor contents were transferred with a pipette to a microcentrifuge tube containing a mixture of zirconia beads (1 mm, 167 μL or ~375 mg; 0.7 mm, 334 μL or ~1,314 mg; 500 μL total) on dry ice. The old tube was rinsed by briefly vortexing with 800 μL MeOH (80% in ddH_2_O), which was added to the beads. This mixture was frozen on dry ice for up to 3 days. Contents were twice homogenized on dry ice for 180 s @1,800 rpm using a MP FastPrep 96 (MP Biomedical; USA) adapted for microcentrifuge tubes, adding dry ice each time. The homogenate was centrifuged at 14k rpm at 4°C for 5 min (18,220x g; centrifuge 5417C; Eppendorf, USA). The supernatant was transferred to a separate microcentrifuge tube and kept on dry ice while the pellets were back-extracted with 500 μL MeOH (80%), homogenized once for 180 s at 1,800 rpm, and centrifuged an additional 5 min. Supernatants from both extractions were combined, then dried to completion in a CentriVap concentrator/CentriVap cold trap −105°C system (Labconco, Kanasas City, MO, USA) for 4–6 h. Pellets for two samples were combined during resuspension in D_2_O (DSS, 1/9 mM) for each condition. Two replicates from each condition were thus pooled and pipetted into 1.7 mm NMR tubes (Bruker).

### Annotation

For each pooled sample representing the anaerobic and the aerobic conditions, noesypr1d, ^13^C-HSQC, total correlation spectroscopy (TOCSY), and ^13^C-HSQC-TOCSY spectra were collected on a 600 MHz Bruker magnet equipped with a 5 mm cryoprobe and an Avance III HD console at the University of Georgia NMR facility. 2D data were processed in NMRPipe (System Version 9.4 Rev 2017.340.17.07 64-bit) and submitted to COLMARm (Bingol et al., 2016) for putative compound identification. After manual inspection, metabolites were assigned a confidence level ranging from 1 to 5, with 5 being the highest. The scale is defined (Walejko et al., 2018) as follows: (1) putatively characterized compound classes or annotated compounds, (2) matched to literature and/or 1D reference data such as HMDB (Wishart et al., 2007) and BMRB (Ulrich et al., 2008) (3) matched to HSQC, (4) matched to HSQC and validated by HSQC–TOCSY [COLMARm (Bingol et al., 2016)], and (5) validated by spiking the authentic compound into sample. Identifications from extracted 1d spectra were manually mapped to real-time *in vivo* noesypr1d data. An additional score was assigned to each mapped compound: 0 (unannotated), 1 (annotated only), 2 (qualitatively assessed), or 3 (relatively quantifiable) in the real-time data. This score depended on number of observed peaks, baseline, peak overlap, and sensitivity. Both metabolite confidence levels are reported in Supplementary Table 1. All raw and processed data files are available at http://www.metabolomicsworkbench.org/ and matching can be run on COLMARm (Bingol et al., 2016) directly.

### Batch Processing in NMRPipe for *in vivo* NMR Data

Parameters were optimized based on agreement between spectra from several time points for a given sample. A custom bash script ran NMRPipe (Delaglio et al., 1995) using the optimized parameters on all spectra for a given sample. This script included all necessary NMRPipe commands for file conversions and NMR data processing. In brief, the following were implemented: line broadening, fast Fourier transform, 0- and 1^st^-order phasing, end removal, and baseline correction using automatic polynomial fitting. All raw data, parameter files and code are available at http://www.metabolomicsworkbench.org/.

### Additional Processing in MATLAB for *in vivo* NMR Data

For each sample, custom scripts were written in MATLAB R2017b (The MathWorks, Inc., Natick, Massachusetts, USA), to load the processed spectra, ppm vectors, and measurement start times from.ft and Bruker acqus files. Spectra were then referenced to DSS semi-automatically, stored as a matrix, and saved as a MATLAB workspace in.mat format. Using custom MATLAB scripts,.mat files from individual experiments were combined into a “sampleData” structure. Metadata (e.g., condition, pulse width, time shift between inoculation and start time) were added to each sample by manual entry or by automated retrieval from the Bruker acqus files for each sample. Spectral ends outside of [−0.5, 10] ppm were removed. The spectral region containing the water signal [4.7, 5] ppm was replaced by zeros. Measurements for time points >11 h were removed in all experiments for consistency. Each spectrum was normalized to its DSS peak intensity as a formal step to allow for relative quantification. Finally, every three spectra were summed starting from the first timepoint for improved signal-to-noise. The resulting structure was saved as a.mat file (~2 Gb). All data and scripts are available at http://www.metabolomicsworkbench.org/ and at https://github.com/artedison/Edison_Lab_Shared_Metabolomics_UGA.

### Relative Quantification of NMR Resonances

A combination of a Gaussian smoothing filter with user-defined sigma in the ppm and time dimensions and peak picking script was used to identify peak maxima for a given region of ~0.5–1 ppm in a given sample, allowing some noise to be picked. Agglomerative clustering based on single linkage of Euclidean distances was then used to cluster the picked points in the chemical shift (ppm), time, and intensity space. Weights for each dimension in the clustering, as well as the number of clusters, were manually optimized for each region and sample. Clusters were quality-controlled by interactive visual inspection. If multiple ridge points existed for the same time, the one with highest intensity was retained. Peak positions at temporal gaps were estimated using linear interpolation between the two closest existing ridge points. Ridges on the smoothed data were mapped to the unsmoothed data for each time point by choosing the maximum within a small window around the peak position obtained from the smoothed data. A window size of 10 indices (~2.9 × 10^−3^ ppm) worked for all but a few ridges, whose optimal mapping windows ranged between 6 and 60 indices (between 1.7 × 10^−3^ and 1.7 × 10^−2^ ppm). All ridges were visually inspected for good tracing, well-defined peaks, and minimal overlap by plotting on real spectra. To combine the trend information from multiple ridges annotated to the same compound, intensities of constituent ridges were scaled such that the ridge means across the time points shared by the highest number of ridges were equal. Lastly, the mean across scaled ridges at each time point was taken, yielding a single composite trajectory for each compound. A tutorial on the use of this workflow is available (Supplementary File 1).

### Titration of a Citrate Standard for Estimation of *in-vivo* pH Changes

A 10 mM solution of citric acid (A104-500; Fisher Scientific, USA) containing 1 mM DSS reference standard was prepared, and 600 μL were added to a 5 mm NMR tube (Norell; Morganton, NC, USA). The pH of the solution was adjusted in-tube in ~0.25 pH increments by addition of 0.5–2 μL volumes of dilutions of concentrated NaOH and HCl and four rounds of inversion and vortex mixing. For each pH point, the pH was measured in-tube using a calibrated accumet AB150 pH meter (Fisher Scientific, USA), then a 1D noesypr1d spectrum was collected (DS = 2; NS = 16) on a 600 MHz Bruker magnet equipped with a 5 mm cryoprobe and an Avance III HD console at the University of Georgia NMR Facility. Data were phased and referenced to DSS in TopSpin (v3.5pl7; Bruker). Custom Matlab scripts were used to obtain the most upfield citrate peak position for each pH. A 3rd-order polynomial was fit to the positions (*R*^2^ > 0.99) and used with the ridge belonging to the same peak to estimate the pH of each culture at each timepoint.

## Results

For all of the experiments reported below, we collected 3 independent biological replicates. The extracted traces from the 3 replicates are displayed. Proper statistical treatments of these time series data are specific to the multiple uses of the data. Different options are presented in the Discussion section, although their application is nuanced and beyond the scope of this manuscript. All data and analysis scripts are available on the Metabolomics Workbench and the Edison Lab GitHub (Sud et al., 2016).

### Isotopic CIVM-NMR Measurements Confirm Unidirectional KIV-to-Valine Flux in ML Cells

Branched-chain amino transferase-1 (BCAT1) is a reversible enzyme, but in most cells the reaction degrades BCAAs and makes branched-chain keto acid (BCKA)s. However, we recently demonstrated that BCKA transamination by the BCAT1 enzyme builds up the BCAA pool in myeloid leukemia (ML) cells, essentially running in the reverse direction (Hattori et al., 2017). When α-keto-isovalerate (KIV; one of the substrates of BCAT1) was ^13^C-labeled, valine (the expected product of BCAT1) containing ^13^C accumulated. Labeled KIV was not observed when ^13^C-labeled valine was supplied, indicating a non-canonical, unidirectional flux from KIV to valine (Hattori et al., 2017). In that study, metabolic fingerprints were acquired via a traditional, labor- and material-intensive sampling scheme involving months of sample preparation and several dozen samples. One reason for the large number of samples in this or similar studies is the biological and technical variation due to sample preparation steps; these factors make it more challenging to compare time-series data without large numbers of replicates. We sought to replicate the result of the original Hattori et al. study using real-time *in vivo* metabolomics.

First, we cultured myeloid leukemia cells as previously described (Hattori et al., 2017), then pelleted and resuspended them in IMDM media without KIV or valine. Working quickly, we loaded the cells into an HR-MAS rotor and added either ^13^C-labeled KIV or ^13^C-labeled valine to make a total volume of ~60 μL, capped the sample, and inserted the rotor into the magnet. We recorded 1D HSQC spectra every 4.2 min while spinning at 3,500 Hz at the magic angle (54.7°) (Beckonert et al., 2010) for three independent replicates of each compound (Supplementary Figure 1). A hole in the rotor cap allowed for gas exchange (Mobarhan et al., 2016).

**Figure 1:**
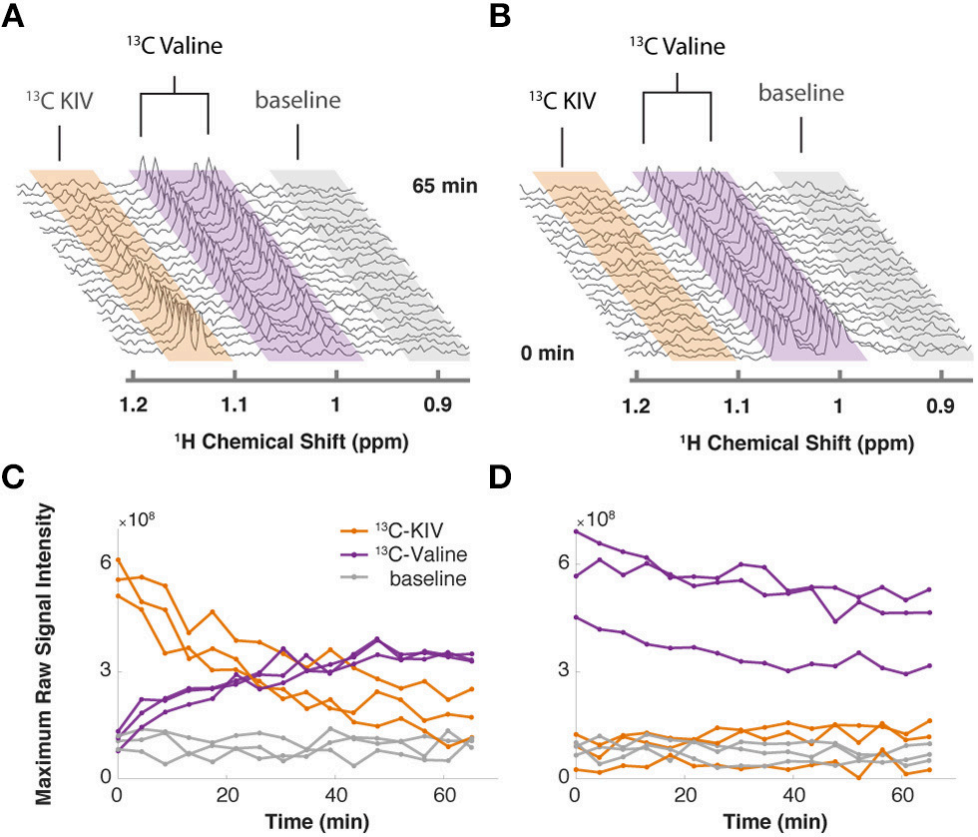
Targeted isotopic CIVM-NMR measurement of metabolic flux in human myeloid leukemia cells. **(A)** ^13^C-labeled keto-isovalerate (KIV) was converted to valine. **(B)** ^13^C-labeled valine was not converted to KIV, confirming unidirectional flux in ML cells. **(C,D)** Relative concentrations over time of ^13^C-labeled KIV (orange) and ^13^C-labeled valine (purple) compared to baseline noise (gray), obtained by taking the raw maximum spectral intensity within each region of the representative experiments in **(A**,**B)**, respectively. Different lines show the data from 3 independent replicates of each experiment.

By monitoring the intensity of the methyl peaks of both KIV and valine, we observed that ^13^C-labeled KIV decreased in intensity and fell close to the limit of detection within about 60 min (Figure 1A). The ^13^C-labeled valine peak grew with an inversely proportional trajectory, providing real-time, *in vivo* evidence of KIV-to-valine conversion. As the reaction rate depended on the concentration of the cells in the rotor, cell density was adjusted to accommodate measurement of the rapid reaction and provide greater detail about reaction kinetics. As reported previously, labeled KIV was not observed when ^13^C-labeled valine was supplied (Figure 1B), showing that the reaction equilibrium heavily favors the production of valine in these cells.

### Untargeted CIVM-NMR Measurements of *N. crassa* Metabolism

**Figure 2:**
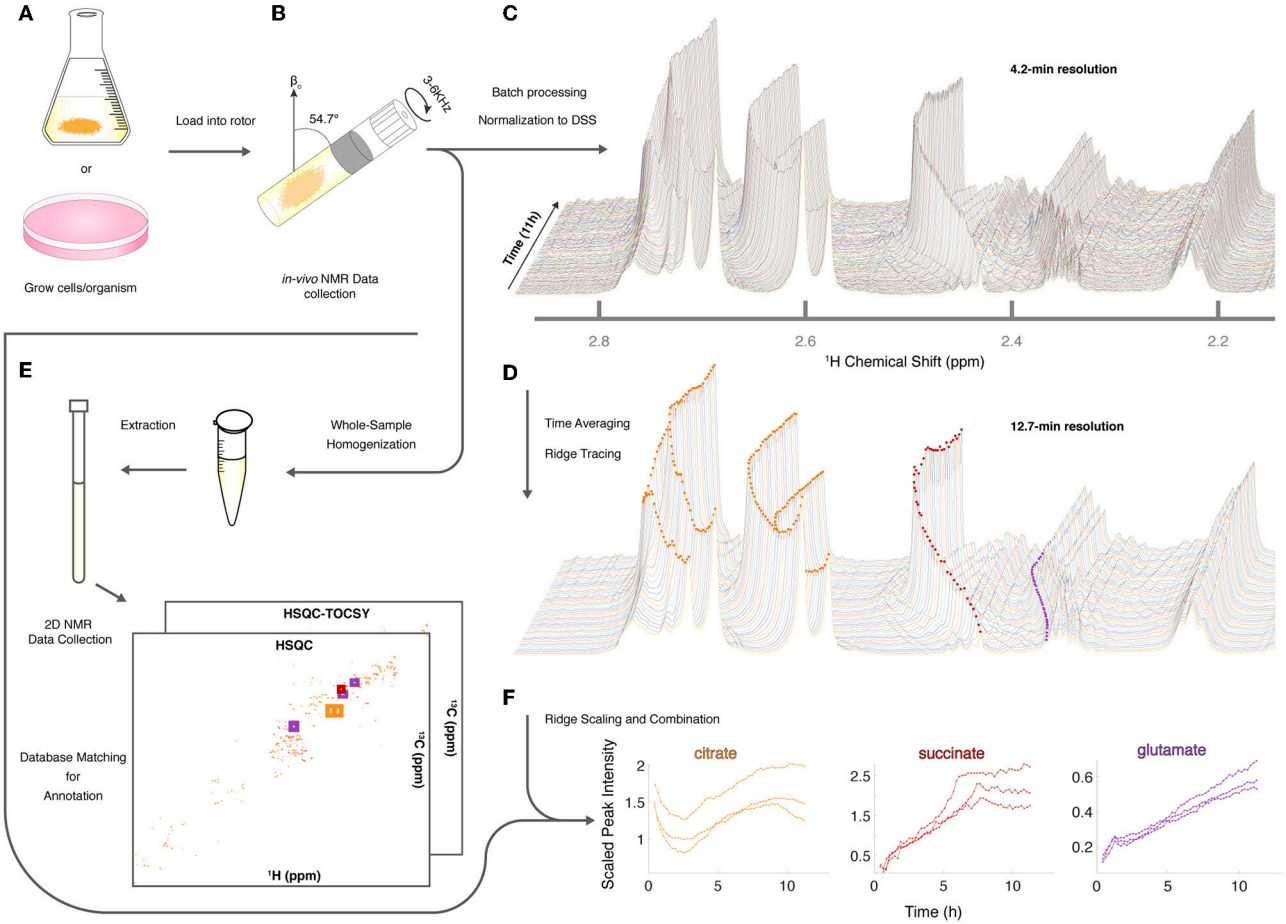
Sample preparation and analysis for CIVM-NMR experiments. **(A)** Samples were first grown to a suitable volume or density in standard media and **(B)** transferred to the HR-MAS rotor (*N. crassa* is shown). Gas composition (e.g., air availability) was altered using a filtered hole or no hole in the cap, and the rotor was spun at the magic angle. NMR data were collected continuously every 4 min over the course of hours, then **(C)** processed and normalized to the DSS reference peak (0 ppm) to yield full-resolution data. **(D)** Every three spectra were time-averaged (summed) for improved S/N, and peak intensities were traced across time using ridge tracing to yield relative quantification of metabolites. **(E)** Following HR-MAS, the rotor contents were homogenized, methanol-extracted, and used for 2D NMR analysis for peak annotation by database matching. **(F)** For annotated metabolites with >1 peak (e.g., citrate), the quantified and annotated trajectories (ridges) for each peak were scaled and combined into a single representative trajectory. Trajectories for each annotated compound in 3 aerobic experiments are plotted to compare time series between biological replicates.

Given the utility of CIVM-NMR for the targeted monitoring of known reactions in mammalian cells, we applied it to the continuous measurement of the metabolic dynamics of the filamentous fungus *N. crassa* over 11 h in both aerobic and anaerobic environments. *N. crassa* is an obligate aerobe but will live under low-oxygen conditions (Slayman, 1965; Slayman and Slayman, 1968; Slayman et al., 1973). We grew *N. crassa* tissue in a nutrient-rich liquid medium (Figure 2A). After 32 h, a piece of tissue with a volume of ~50 μL was taken from the main mycelial mass, rinsed, and put into a 4-mm HR-MAS rotor with fresh media. The rotor was sealed with a cap with a hole filtered with rayon culture tape punches (“aerobic”; Mobarhan et al., 2016) or no hole (“anaerobic”), placed in the HR-MAS probe, and spun at 6,000 Hz at the magic angle for the duration of each experiment (Figure 2B). Each individual scan of a standard noesypr1d experiment took ~3.97 s. Scans were recorded and summed continuously, and free induction decays (fids) were written to a file once every 64 scans, establishing our shortest temporal resolution at 4.23 min (Figure 2C). After data acquisition, properly phased and Fourier-transformed frequency-domain data were again added together sequentially to increase the signal-to-noise ratio (S/N), resulting in 12.7-min temporal resolution for all downstream analyses (Figure 2D). The organism was assessed for survival after each experiment (ranging between 11 h to 4 days). In every case (*n* = 9), mycelia did not sediment, were intact, and grew significant hyphae within hours of being placed on standard nutrient agar after the experiment (Supplementary Figure 2). Thus, *N. crassa* survived the CIVM-NMR experiments and could be used in downstream experiments or processing steps (Figure 2E). Custom shell scripts allowed for batch processing of NMR data (Figure 2D) using NMRPipe (Delaglio et al., 1995). Normalizing to the stable 1 mM DSS reference resonance (0.0 ppm) allowed for relative comparison of peak intensities across time points and samples.

**Figure 3:**
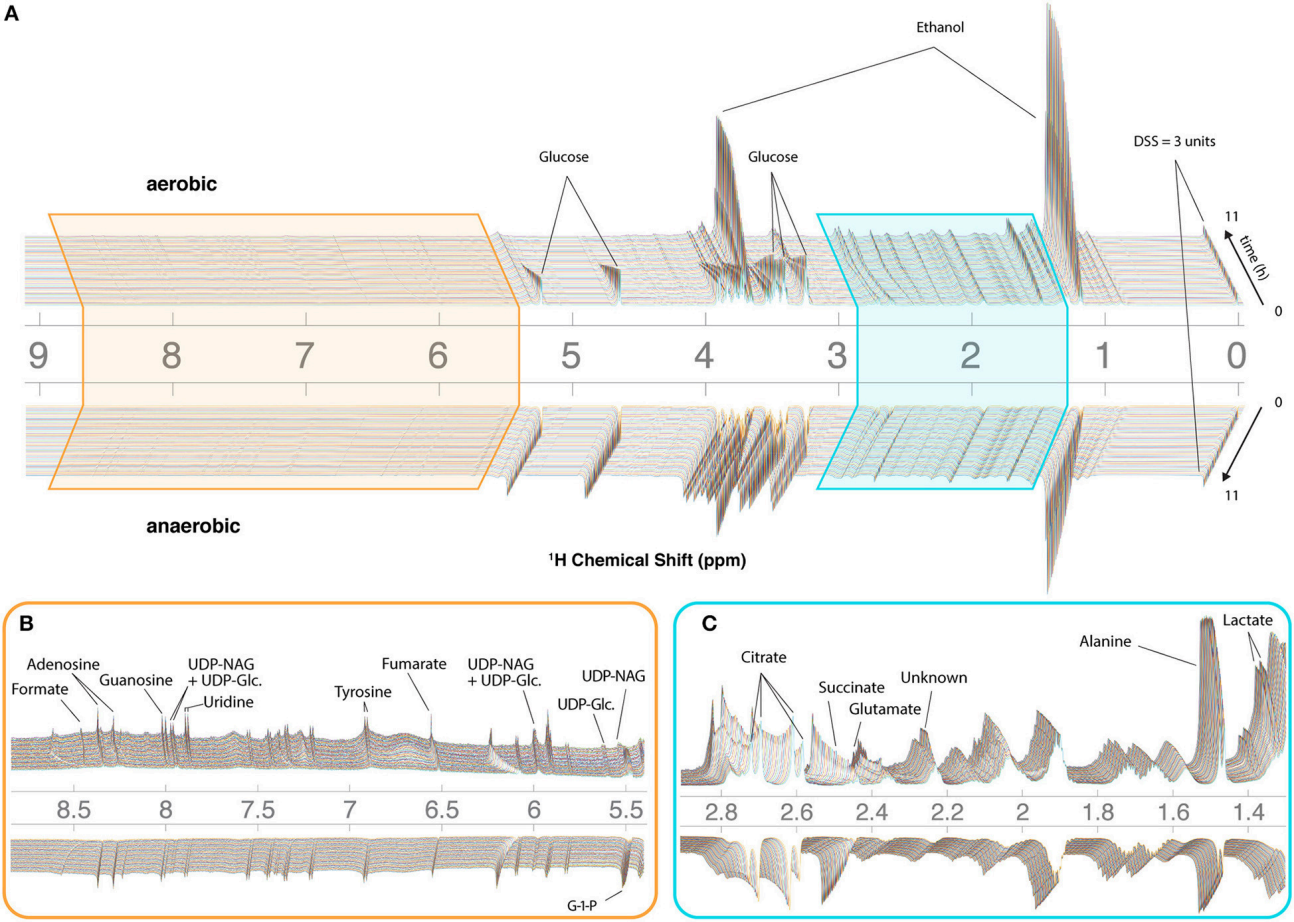
CIVM-NMR measurements of *N. crassa* metabolism under aerobic and anaerobic conditions. ^1^H NMR data for one aerobic replicate (top) and one anaerobic replicate (bottom) plotted interactively as a “mirror plot” for direct comparison between conditions by peak height and position at a given time. To improve the S/N, data were analyzed at 12.7 min resolution. Annotations are shown for select peaks of interest for **(A)** the entire spectrum, and expansions of **(B)** the aromatic region and **(C)** the aliphatic region. Several peaks change position and intensity over the course of the experiments. UDP-NAG, UDP-N-Acetyl Glucosamine; UDP-Glc, UDP-Glucose; G-1-P, Glucose-1-Phosphate.

To assist with annotation and compound identification, the organism and media were removed at the end of each run, bead-homogenized, and extracted in MeOH (80%) (Figure 2E). Combined supernatants for representative samples were analyzed using 2D ^13^C-HSQC and HSQC-TOCSY NMR experiments, and the data were matched to an NMR metabolomics database using COLMARm (Bingol et al., 2016). Resulting putative identifications were manually assigned confidence scores as described previously (Walejko et al., 2018). We mapped 34 metabolites with high confidence scores onto the real-time *in vivo* spectra of *N. crassa* (representative annotations, Figure 2F), including multiple amino acids and metabolites involved in the TCA cycle, glycolysis, and fermentation (Figures 3A–C, Supplementary Table 1). Several metabolites overlapped with those found in a previous NMR study in *N. crassa* (Kim et al., 2011). We created MATLAB functions for visualization of time series data for samples individually (Figures 2C,D) or as interactive mirror images (Figure 3). We found that the latter approach facilitated comparison between samples, revealing several differences in metabolism between the aerobic and anaerobic conditions (Figure 3) that were reproduced in replicate samples (Supplementary Figure 3).

**Figure 4:**
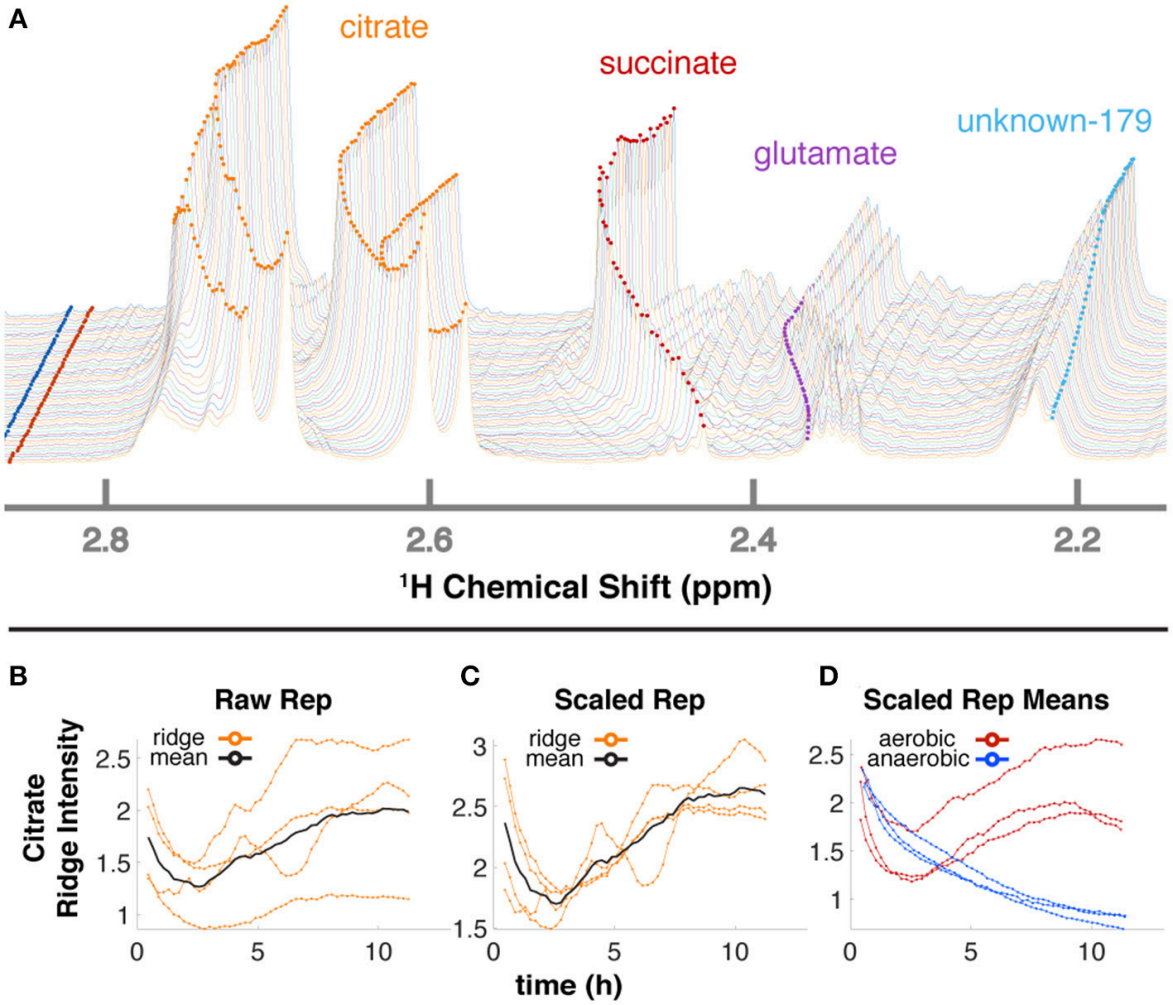
Ridge tracing produces concentration dynamics of metabolites. **(A)** Multiple traced ridges for a single aerobic replicate. Peak maxima at each time point were located using a peak-picking algorithm that includes an adjustable Gaussian filter. Maxima were connected to form ridges along the time dimension using a single linkage hierarchical agglomerative clustering based on Euclidean distances between the points in chemical shift, time, and intensity space. Metabolites typically have several characteristic NMR peaks, e.g., the 4 orange ridges in citrate **(A)**. A simple time-wise average represented by the black line in **(B)** only gives the average intensity over time but loses valuable information on actual dynamic trends. To more accurately extract trends for a particular metabolite, we first integrate each peak in that metabolite over time to obtain its mean value. Then, each peak trajectory is scaled by ratio of the highest mean to its own mean, yielding the 4 orange lines in **(C)**. The mean of these trajectories is shown in black in **(C)** and represents the relative concentration over time for that metabolite in that replicate. The 3 aerobic (red) and 3 anaerobic (blue) replicates for citrate are shown in **(D)**.

The 34 compounds that were mapped to *in-vivo* data were assigned a second confidence score for quantifiability. For 21 highly scoring metabolites (Supplementary Table 1), we obtained relative quantification (Supplementary Figure 4) by tracing peaks across time with a ridge-tracing algorithm (Figures 2D, 4A). With our current algorithm that is limited to peaks with low overlap, we traced over 170 peaks across all of our spectra, including ~150 that are currently un-annotated. We combined the information from ridges of sufficient quality when assigned to the same compound (Figures 4B–D), leveraging the information about compound concentration from multiple measurements.

### Glucose-Dependent Changes in pH

NMR chemical shifts are sensitive to pH and metal ion content (Tredwell et al., 2016; Ye et al., 2018), typically requiring peak alignment algorithms that are prone to creating artifacts. The positions of peaks clearly changed across time in our data (Figures 3B,C, 4A), particularly in the aerobic samples. Because these changes were monitored continuously, peak identity across time was unambiguous, eliminating the need for alignment and facilitating annotation and quantification even as changes in peak position affected overlap with other peaks. Changes in peak position for organic acids in our samples were compared with reported titration curves (Koczula et al., 2016; Tredwell et al., 2016; Ye et al., 2018), in-house titrations for citrate (Supplementary Figure 5), and Bruker AssureNMR software (Bruker Biospin, USA; Supplementary Table 2) to estimate pH of the sample at each timepoint. Our data indicate that the pH of the aerobic cultures began at 6.2–6.4, then dropped to 5.2–5.4 with glucose consumption. Furthermore, this acidification reversed after glucose depletion at 6–7 h, and pH increased to 5.5–5.7 by the end of our experiments. In the anaerobic samples, the pH decreased from 6.2–6.3 to 5.7–5.9. Although we did not perform high-resolution titrations for glutamate, succinate, and fumarate, their reported shifts were consistent with the trends for citrate (Supplementary Table 2).

### Activation of Central Carbon Metabolism in Aerobic Conditions

**Figure 5:**
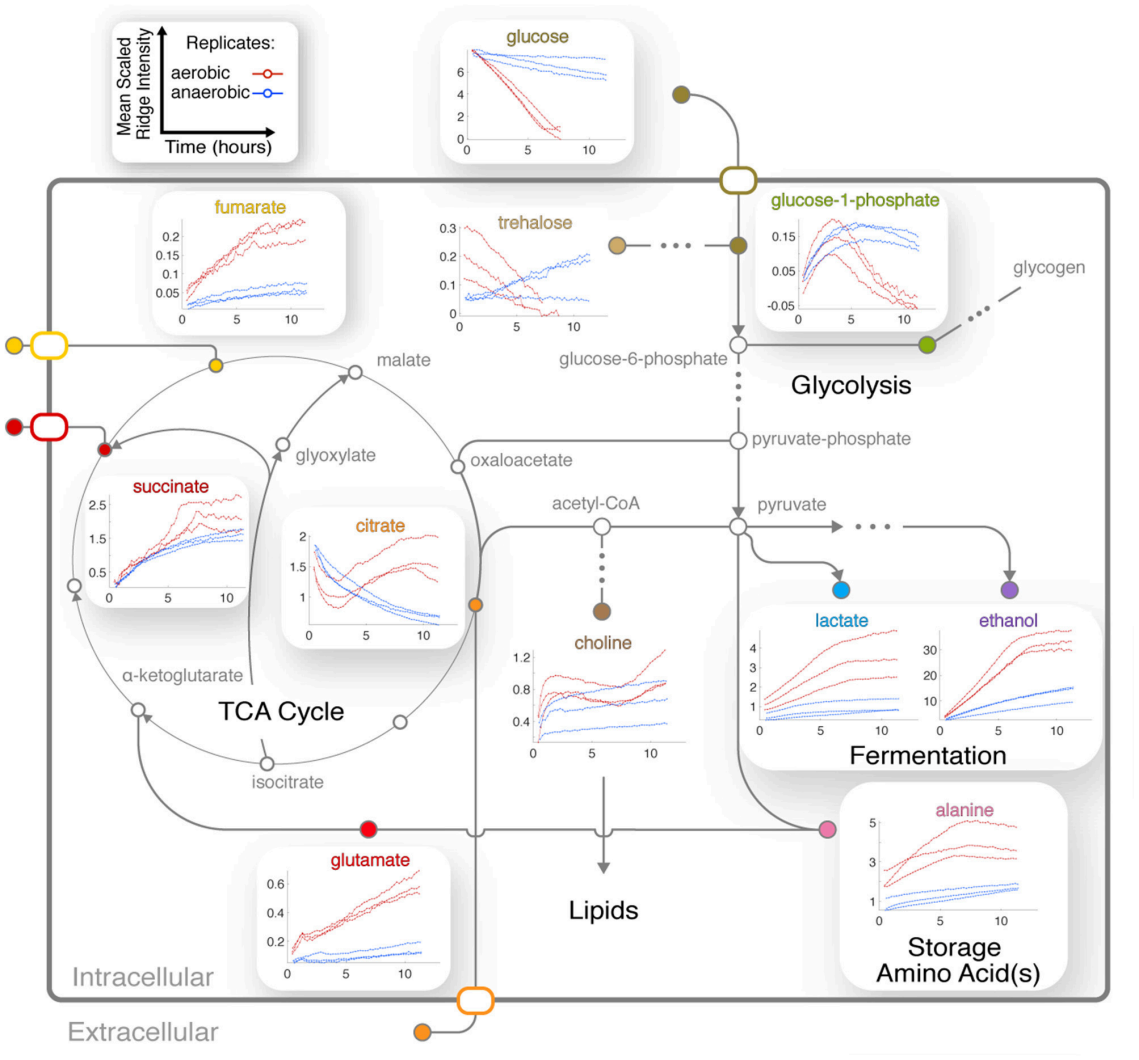
Integration of central metabolic pathways. Arrows correspond to one or more reactions, and nodes correspond to metabolites (Dreyfuss et al., 2013; Kanehisa et al., 2016). Nodes are filled for observed metabolites. Plots show the means of scaled peak/ridge intensities for a given compound in a given replicate over traceable times, where red and blue trajectories represent aerobic and anaerobic conditions, respectively. Arrows indicate typical reaction directions. The glyoxylate cycle is shown as a shunt through glyoxylate embedded in the TCA cycle.

Four TCA cycle metabolites were detected in our experiments (Figures 3B,C). Fumarate and succinate increased in the aerobic condition, and both accumulated slightly faster around 6 h following glucose depletion and remained abundant (Figure 5). Standard replicate averaging with extracted samples at different times would average out much of this detail. In contrast, in low oxygen levels in the anaerobic sample, we observed a slight reduction in succinate compared to a much greater reduction in fumarate. Succinate levels in the aerobic condition are comparable to those in the anaerobic condition, while fumarate accumulates much more in the aerobic condition (Figure 5). Finally, citrate was abundant in the aerobic condition and followed a complex trend, while malate was observed in endpoint extracts (Supplementary Figure 6A). Similar trends with lower rates were observed in the anaerobic samples, except for differences in citrate and glucose-1-phosphate (G-1-P) (Figure 5).

### Interplay Between Amino Acid, Central Carbon, and Nitrogen Metabolism

The dynamics of glutamate were different between aerobic and anaerobic conditions, with a much greater increase of this key amino acid in aerobic conditions (Figure 5). Glutamate accumulates while synthesis of glutamine is repressed in *N. crassa* in nitrogen-sufficient conditions (Kanamori et al., 1982). We could not annotate glutamine with confidence because of overlap (Supplementary Table 1B). However, resonances consistent with glutamine increased after ~3 h (Supplementary Figure 6B), indicating potential nitrogen insufficiency in the aerobic culture. Arginine levels correspond to those of glutamate in the aerobic condition (Supplementary Figure 4).

Trends for alanine (Figure 5) and an unknown in the aliphatic region (Figures 3C, 4A) were very similar to that of ethanol and lactate (Figure 5), indicating that their metabolic fluxes are closely dependent on intermediates or energy produced by glycolysis and fermentation. This hypothesis is supported by the fact that alanine is synthesized from glutamate and pyruvate by alanine transaminase (Kanamori et al., 1982; Radford, 2004). Glutamate levels increased and were unaffected by glucose, but alanine first accumulated and then decreased upon glucose depletion (Figure 5).

### Complex Trends Reveal Dynamics Between Energy Storage and Cell Wall Synthesis Pathways

CIVM-NMR data revealed significant changes that preceded glucose depletion at ~6 h for compounds such as citrate, choline, adenosine, and valine, which all had similar trends in the aerobic condition (Figure 5). Citrate decreased at the start of all experiments. Under aerobic conditions it began to accumulate again around 2.5 h and surpassed initial levels, while in anaerobic conditions it decreased at an exponential rate to a very low amount (Figure 5). Glucose-1-phosphate (G-1-P) is converted to UDP-glucose by the enzyme UTP-glucose-1-phosphate uridylytransferase. UDP-glucose, in turn, is a precursor in *N. crassa* cell wall and glycogen biosynthesis. Levels of G-1-P increased in the aerobic samples until around 3 h then decreased, while UDP-Glucose was also observed but not quantified due to low concentrations. G-1-P accumulated to comparable levels in both conditions, but it remained observable for the duration of the experiments in the anaerobic samples. The primary chitin cell wall building block UDP-N-acetylglucosamine (UDP-GlcNAc) (Milewski et al., 2006) increased in only the aerobic cultures (Figure 3, Supplementary Figure 6C), although overlap and low intensity prevented quantification.

### Glucose Flux Exposes Dynamics Between Glycogen, Glucose, and Fermentation

In the experiments reported above (Figures 3, 5), glucose and trehalose were consumed within the first 6 h, while ethanol and lactate were produced under aerobic conditions. To confirm flux from glucose through these pathways, we conducted an interleaved time-series measurement of both ^1^H 1D and ^13^C-HSQC 1D data after feeding uniformly labeled ^13^C-glucose to starved *N. crassa* (Supplementary Figure 7). These measurements were very informative, as we could not only track the flux of ^13^C over time but also record a combination of both ^13^C-labeled and unlabeled metabolites in the ^1^H 1D data. Furthermore, the ^13^C metabolites were still coupled in the ^1^H 1D dataset, causing predictable and symmetric peak splitting patterns that allowed us to easily distinguish protons attached to ^12^C and ^13^C.

**Figure 6:**
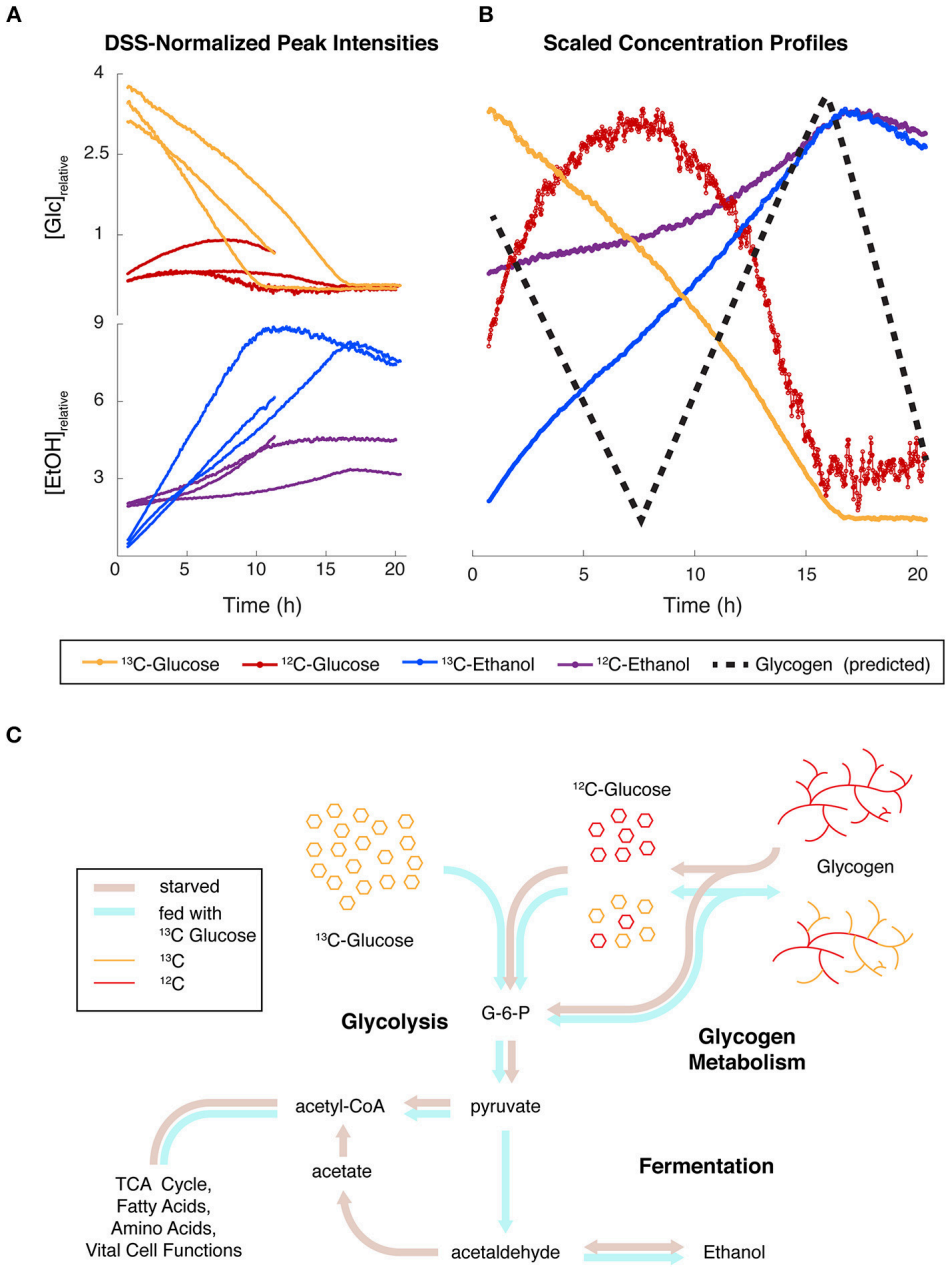
Simultaneous monitoring of carbon isotopes reveals convergence of major glucose fluxes from different origins. **(A)** Relative concentrations for Glucose (Glc) and Ethanol (EtOH) containing ^12^C and ^13^C in *N. crassa* cultures fed with ^13^C-labeled glucose (*t* = 0 h) after 2 h of starvation. Protons covalently attached to ^12^C and ^13^C were differentiated in noesypr1d experiments by ^13^C-induced splitting. Three independent replicates are shown. One replicate was only recorded for 11 h. **(B)** Relative concentrations scaled by the maximum of each trajectory of one replicate highlight the relationships between trends. A hypothesized glycogen trajectory is plotted as a black dotted line. **(C)** Hypothesized fluxes through glycolysis, glycogen metabolism, and fermentation under starved (brown arrows) and fed (blue arrows) conditions.

The normalized intensities from 3 independent replicates of both isotopes (^13^C or ^12^C) of both glucose and ethanol clearly show that the intensities of both compounds are largely mirroring each other, but the trends are different between isotopes. The same compounds from a single replicate were scaled for detailed comparison, and we superimposed a dashed line showing predicted glycogen levels (Figure 6B), which were not measured in this study. Figure 6C is an overview of the major pathways (glycogen, glycolysis, fermentation, and TCA, etc.) that are implicated in this experiment. The different colors of thick lines indicate proposed fluxes under starved or fed conditions.

## Discussion

### Overall Benefits and Practicalities of Continuous Metabolic Measurements

CIVM-NMR is an approach to monitor metabolic dynamics in cells and whole microorganisms. An uninterrupted, high-resolution time series of NMR data allows observation of rapid and reproducible metabolic events. In contrast, using traditional studies with different replicates for each time point, the biological and technical variation often obscure details of dynamics. The lack of extraction removes a major source of technical variation found in typical MS and NMR metabolomics workflows, and CIVM-NMR is fast and simple to implement once conditions are optimized. For example, the original results on BCAA flux in myeloid leukemia cells from Hattori et al. took months of sample prep and data collection but were reproduced here with real-time resolution (Figure 1) in a few days. We did not need to adapt the culture media (Link et al., 2015) or embed the cells (Koczula et al., 2016) to get these results. Additionally, the combined rate of uptake and conversion of valine could be measured with precision, where measurements at only a few time points were taken previously.

In comparison to mass spectrometry-based methods, NMR has relatively low sensitivity. However, it is quantitative and reproducible, and conventional NMR cryoprobes allow routine ^1^H detection of compounds at concentrations as low as about 5 μM. HR-MAS probes that are utilized in CIVM-NMR are less sensitive, but the temporal dimension of CIVM-NMR data allows for more confident assignment of peaks by monitoring their continuous change. By taking advantage of this unique property of CIVM-NMR data, we detected peaks as low as ~24–62 μM ^1^H (Supplementary Figure 8). This sensitivity is ideal for observation of the major sources, sinks, and bottlenecks of metabolism in an organism or cells (e.g., for metabolic engineering). For instance, absolute quantification of 103 metabolites in *E. coli* by LC-MS/MS revealed intracellular concentrations ranging from 0.13 μM to 96 mM. Of these, 61 were found in concentrations of 100 μM or higher (Bennett et al., 2009), placing them well within the detection limits of CIVM-NMR.

Only 20–70 μL of sample is needed with no sample preparation to yield an entire time series of abundant metabolites, and the sample can be used in downstream *in vivo* or chemical analyses following NMR data collection. These factors make CIVM-NMR ideal for scarce samples that would not otherwise be possible to study by time-series metabolomics (Sefer et al., 2016). With an internal rotor radius of 1.4 mm spinning at 6,000 Hz, our samples experienced up to 200,000x g of acceleration. As sedimentation was not observed, it is possible that a low relative density of *N. crassa* mycelia compared to the media may have resulted in a lower effective radius of rotation. While some samples, including the leukemia cells in Figure 1, are less stable at high spinning rates, microorganisms such as *E. coli* and *S. cerevisiae* can grow under different amounts of hypergravity, even with cellular and organellar sedimentation (Deguchi et al., 2011). Furthermore, methods have been developed to obtain HR-MAS data with slow spinning (Mobarhan et al., 2017), which could allow monitoring in ~1,500x g or less. The lack of perfusion and a limited sample volume are both factors that need to be considered with regard to nutrient depletion and waste accumulation.

### Data Analysis and Modeling

Identification of spectral features and deconvolution of overlap are challenging in CIVM-NMR, as with any NMR or LC-MS metabolomics study. However, temporal continuity clearly provides information that is helpful in addressing these problems. For instance, individual multiplet structures are preserved with changes in chemical shift, even across times during which there is substantial overlap with other peaks. Using this information, we can confirm the presence of both compounds at those time points. This same information will be useful for spectral deconvolution methods yielding relative or absolute concentrations, a major goal in NMR metabolomics data analysis.

Replicates of dense, continuously repeated measurements on the same sample offer other benefits, such as separation of inter-and intra-sample noise (Sefer et al., 2016) that would be eliminated by taking time-wise averages or employing standard error analysis. Statistical treatment of CIVM-NMR data should leverage these advantages and should be approached from different perspectives depending on the goals of the analysis. Here, we demonstrated the utility of plotting relative concentrations in a pathway context for interpretation of broad metabolic trends. These trajectories could also be clustered under functional data analysis (FDA) or frequency domain analysis, which are more systematic mechanisms for identifying patterns among metabolites through time. Additionally, statistical tests can be formulated through these approaches to compare metabolic status between conditions (Leng and Müller, 2005; Febrero-Bande and de la Fuente, 2012; Aghabozorgi et al., 2015). A more comprehensive metabolic analysis can also be done in a kinetic modeling framework using ensemble modeling. By explicitly modeling reactions, enzyme parameters can be statistically analyzed; these parameters are inaccessible for implicit methods mentioned above. Lastly, a kinetic modeling framework will not only yield meaningful confidence intervals for these trajectories, but it will also produce testable predictions based on existing data (Yu et al., 2007).

Our data underscore the need for accurate and experimentally-based kinetic models of metabolism. We achieved temporal resolution as high as 1 min in the ^13^C labeled experiments presented here by using fewer scans before saving fids. While this comes with a cost in signal-to-noise ratio, we can recover signal-to-noise by employing moving averages. If noesypr1d experiments are not interleaved, 22-s resolution is easily achieved. Thus, CIVM-NMR provides a unique opportunity for probing rapid flux changes and allosteric regulation (Link et al., 2013) with kinetic models (Link et al., 2014, 2015) for abundant metabolites. Each replicate can be formulated as a single, complete model with different initial conditions, which is more appropriate for kinetic modeling applications than a time series of averages. Previous real-time methods have equal or greater temporal resolution at the expense of disadvantages such as being destructive (Link et al., 2014), limitation to cell suspensions (Link et al., 2014; Koczula et al., 2016), primarily measuring the media (Koczula et al., 2016; Sengupta et al., 2016), measuring broad classes of metabolites (Kang et al., 2012; Shalabaeva et al., 2017), or having combined biological and technical variance. CIVM-NMR minimizes noise by eliminating sampling and extraction variance. Batch effects for each replicate are eliminated since all experimental and NMR parameters are consistent across timepoints. Analytical drift is eliminated because the detector never contacts the samples, and the sample is not perturbed by measurement. These factors in turn facilitate optimization of modeling parameters (Ghasemi et al., 2011).

### Cell Viability

HR-MAS NMR experiments apply strong centrifugal forces and do not allow for easy media exchange during growth. A perfectly reasonable question is whether cells are viable during and after the experiment, as opposed to simply a collection of enzymes that can still function. In some cases, cells will be too delicate to analyze, even at low spinning speeds. However, for the relatively delicate human leukemia cells used for Figure 1, we had strong agreement of flux from KIV to valine in both the prior studies using separate extracted samples (Hattori et al., 2017) and the CIVM-NMR real-time measurements reported here. However, to get these to work we needed to lower the spinning speed to 3,500 Hz and only measure for 1–2 h.

For *N. crassa*, there are several lines of evidence that support the claim that the organism was viable during and following the experiment:

1. ***N. crassa*** **taken from the HR-MAS experiments can inoculate a standard lab culture**, with the expected circadian growth patterns (Supplementary Figure 2). This would not occur if all the cells were dead, but it could happen with only a fraction of the cells were alive.
2. **Glycolysis, glycogen degradation, and fermentation all are coordinated** (Figure 6). Our ^13^C-glucose labeling experiments provide extensive evidence for cell viability. For these experiments, *N. crassa* was starved and had only a small amount of EtOH available in the media as a carbon source. These conditions activate glycogen degradation, which is exergonic and releases G-1-P and glucose directly in an approximate 9:1 ratio (Voet and Voet, 2011). Glycogen synthesis occurs during high rates of growth in *N. crassa*, and wanes during slow growth (Brody and Tatum, 1967; de Paula et al., 2002; Virgilio et al., 2017). When we added ^13^C-glucose in aerobic conditions, we observed a surprising buildup of ^12^C-glucose without a corresponding decrease in any other ^12^C peaks, suggesting that glucose may have been released from glycogen stores.
  This accumulation may be expected under glycogen degradation because the conversion of glucose to G-6-P by hexokinase competes with the conversion of G-1-P to G-6-P by phosphoglucomutase on several fronts. First, the hexokinase reaction is a classic case of product inhibition (Voet and Voet, 2011), and relies on ATP (Dreyfuss et al., 2013), which is presumably in high demand under starvation. The phosphoglucomutase reaction, on the other hand, produces the hexokinase product/inhibitor G-6-P, it only consumes G-1-P, and it is not inhibited by buildup of glycolytic intermediates. Next, glycogen degradation only produces one molecule of glucose on average for every nine molecules of G-1-P. As the stoichiometries of the hexokinase and phosphoglucomutase reactions are equivalent, glycogen degradation results in much higher flux through G-1-P. This flux would enhance G-6-P inhibition of the former reaction, as well as consume more ATP further along in glycolysis. Finally, higher levels of ^13^C glucose inside the cell would outcompete the lower levels of ^12^C glucose derived from glycogen; these would then be consumed at lower overall rates proportional this competition.
  If ^12^C and ^13^C glucose formed a common pool above glycolysis, the combination of the effects above had to be quite strong to allow the production of ^12^C glucose to overcome consumption by hexokinase. ^12^C glucose continued to accumulate for about 5 h post-feeding, indicative of a lag time to downregulate glycogen degradation. Both ^13^C and ^12^C glucose then fell below limits of detection contemporaneously within each replicate. This suggests that both isotopes of glucose made up a common pool which was fed by external ^13^C glucose uptake and ^12^C glycogen degradation. This, in turn demonstrates a coordinated cellular process. In Figure 6B we provide a prediction of glycogen levels that we will test in future studies.
3. **Isotopic species of EtOH and glucose are metabolically coupled**. Figure 6 is also informative from the perspective of EtOH produced. We can monitor 2 pools of EtOH in this ^13^C-glucose experiment. The ^13^C-labeled EtOH rises immediately and is largely inversely proportional to the ^13^C-glucose consumed. This suggests that the added glucose greatly exceeds the core metabolic functions of the cell, and the rest is fermented. ^12^C-EtOH also inversely mirrors ^12^C-glucose levels, showing the functional coupling between these species. After depletion of both ^13^C- and ^12^C-glucose, both isotopes of EtOH levels begin to decrease, because *N. crassa* can utilize EtOH as a carbon source to biosynthesize acetyl-CoA (Figure 6). We observe small differences in rates of consumption of the different isotopic forms of EtOH, which might result from different fractions of ^12^C/^13^C EtOH inside and outside of the cell. The inner pool would be consumed more rapidly, because the outer pool needs to be transported into the cell. We need to conduct more experiments to verify this hypothesis.
4. **Functional mitochondria are required** to understand large metabolic differences between aerobic and anaerobic conditions (Figures 3, 5). Most striking are the different levels of fumarate produced in each condition (Figure 5). This can be explained by the fact that conversion from succinate to fumarate depends on oxygen reduction in the electron transport chain (Dreyfuss et al., 2013; Kanehisa et al., 2016). Glyoxylate cycle activity can occur in anaerobic conditions (Wayne and Lin, 1982; Rude et al., 2002) and yields succinate and malate without fumarate as an intermediate. *N. crassa* does not survive on citrate as a sole carbon source (Wolfinbarger and Kay, 1973), and to our knowledge extracellular citrate utilization has not been reported for *N. crassa*. However, citrate levels were observed well below the initial amount present in the media alone (9.74 mM) in both conditions, strongly indicating that external citrate was consumed in both experiments. Isotopic labeling experiments will more directly test this hypothesis. Furthermore, the aerobic conditions show much larger shifts in pH over the course of the experiment (Figure 3). Maintenance of characteristic differences in pH is well-accepted between organelles, the cytoplasm, and the extracellular milieu (Magnuson and Lasure, 2004; Casey et al., 2010; Bencina, 2013). Filamentous fungi including *N. crassa* (Vrabl et al., 2012) secrete large amounts of organic acids such as citrate, fumarate, and succinate, to acidify their extracellular environment (Magnuson and Lasure, 2004; Kubicek et al., 2010; Dörsam et al., 2017), and the two latter acids are taken up by carbon-limited *N. crassa*, with maximal uptake occurring around pH 5.5.
5. **Glutamate stores are maintained**. Glutamate is produced from arginine degradation (Voet and Voet, 2011); for instance, arginine has been reported as an abundant amino acid in extracted samples of actively growing *N. crassa* cultures (Kanamori et al., 1982; Kim et al., 2011) and is thought to be catabolized to glutamate during conidiation (Kim et al., 2011). Arginine and glutamate both accumulate more in our aerobic samples (Supplementary Figure 4), indicating a potential sufficiency. Alanine is derived from glutamate when glucose was depleted in the aerobic conditions, alanine levels began to decrease but glutamate levels continued to increase. Alanine is derived from glutamate and pyruvate, and therefore we conclude that alanine synthesis was limited by a lack of pyruvate from the glucose depletion. Glutamate levels are maintained during starvation (Voet and Voet, 2011), and Kanamori et al. (1982) suggested that alanine serves as a storage for pyruvate and nitrogen *via* glutamate in favorable conditions (Kanamori et al., 1982). Therefore, the observed decrease in alanine suggests that it was utilized for pyruvate and glutamate when glucose concentrations were low in the aerobic condition (Figure 5). Our data therefore support glutamate as a hub between central carbon and amino acid pathways and confirms the maintenance of glutamate stores even under starvation.
6. **Pyruvate and acetyl-CoA both serve as crossroads between major energy metabolites and lipids**. Although we did not observe pyruvate and acetyl-CoA directly, most accumulating metabolites in pathways emanating from pyruvate exhibited strikingly similar trends (Figure 5), suggesting flux through pyruvate. Curiously, citrate and choline did not follow this pattern, indicating activity from pathways that consume and replenish their pools. However, the rates of change of these metabolites were clearly opposed in both aerobic and anaerobic samples. This opposition suggests that flux from acetyl-CoA was being channeled differentially between citrate and choline synthesis and demonstrates a carbon and energy exchange between central metabolism and lipid precursors (Markham et al., 1993). Prior work has indicated that under low oxygen or glucose depletion *N. crassa* cells become vacuolated (Slayman et al., 1994; Slayman and Potapova, 2006). The synthesis of membranes for the vacuoles and their membranes under anaerobic conditions would explain the rise in choline. A concordant decrease in G-1-P at ~3 h may indicate a shift of carbon flux to glycolysis from glycogen, caused by sensing of extracellular glucose levels (Wang et al., 2017) or limitations of glycogen capacity. Glucose conversion to G-6-P (Glucose 6-phosphate) is the first step of glycolysis (Voet and Voet, 2011), which was clearly active in the first stages of our aerobic condition (Figure 5). High levels of G-6-P drives its conversion by phosphoglucomutase to G-1-P (Voet and Voet, 2011), which is converted by UDP-glucose pyrophosphorylase and UTP hydrolysis to the direct glycogen precursor UDP-glucose (Voet and Voet, 2011). The latter is the rate-limiting step in glycogen synthesis, which is an endergonic process. If G-6-P levels were high and flux were shunted to glycogen, high levels of G-1-P would be expected.
7. **Cell wall synthesis, glucose, and oxygen are coordinated**. UDP-GlcNAc is synthesized via the unidirectional Leloir pathway (Milewski et al., 2006), and the only known uses for UDP-GlcNAc in *N. crassa* are chitin/cell wall biosynthesis and UDP-GalNAc production (Edson and Brody, 1976; Milewski et al., 2006). Filamentous fungi such as *N. crassa* produce chitinases (Patil et al., 2000) and could utilize these for autolysis under stress conditions. However, if an increase in UDP-GlcNAc indicated cell wall degradation (i.e., due to stress or autolysis), those resonances would be expected to increase in the anaerobic condition; however, they were barely detected (Figure 3, Supplementary Figure 6C). Curiously, a recent study suggested that *N. crassa* utilizes alternative chitin catabolism pathways that would not result in increased GlcNAc-derived UDP-GlcNAc (Gaderer et al., 2017). Considering the above dynamics, we conclude that resources were allocated between energy storage and cell wall synthesis pathways in glucose-rich conditions.

CIVM-NMR is complementary to a number of other omic assays, such as transcriptional profiling (DeRisi et al., 1997), protein-DNA interactions assays (Ren et al., 2000), protein profiling by ICAT (Gygi et al., 1999), and protein-protein interaction assays (Walhout et al., 2000). The technique provides a phenotypic readout of the most dynamic components of the system in real time. In conjunction with methods at the transcript or protein level, CIVM-NMR closes the gap in the iterative process of prediction and measurement and enables integrated approach to identifying genetic networks (Battogtokh et al., 2002; Yu et al., 2007) and model-guided discovery (McGee and Buzzard, 2018). In this way, CIVM-NMR significantly adds to the goal of systems biology by allowing full data integration from genes to metabolites (Ideker et al., 2001).

## Author Contributions

MJ, AE, and JA designed the project. MJ, YW, and AE wrote the manuscript. MJ and YW wrote analysis scripts and analyzed the results. MJ prepared *N. crassa* samples and extracts. MJ, AE, and JG performed *in vivo* metabolite measurements and processed the data. FT collected and annotated 1D and 2D experiments on extracts. TI, AH, and JG designed and performed *in vivo* carbon-labeled measurements on human cells.

### Conflict of Interest Statement

The authors declare that the research was conducted in the absence of any commercial or financial relationships that could be construed as a potential conflict of interest.
