# Supplementary material for: Continuous in vivo Metabolism by NMR
Source: Front Mol Biosci. 2019 Apr 30;6:26. doi: 10.3389/fmolb.2019.00026 (PMC6502900; doi:10.3389/fmolb.2019.00026)
Supplement: Supplementary file 2 [file Table_2.pdf]

Supplementary Material

Compound	Timepoint 1		Timepoint 89		Timepoint 157		Peak(s) Used (ppm)
	Lower Bound	Upper Bound	Lower Bound	Upper Bound	Lower Bound	Upper Bound	
Citrate	5.5	6	5	5.5	5	5.5	2.8-2.5
Glutamate	7.5	8	4.5	5	5	5	2.4-2.3
Succinate - d2O	5.6	7	--*	5.6	--	5.6	2.4-2.5
Succinate - surine	5.5	6	4	4.5	4.5	5	2.4-2.5
Fumarate	8	--	4.5	5	5	5.5	6.45-6.6
Alanine	8	--	4	--	4	--	1.35-1.4

Supplementary Table 2. Estimation of *in vivo* pH using various organic acids using AssureNMR for the aerobic sample (**Figure 2-4**). Because of limitations on pH resolution in the AssureNMR BBioRefCode database, only lower and upper bounds for pH estimates are shown. Succinate was present in two different sample matrices in the database; both results are shown. The trend of decreasing pH until the point of glucose depletion (Timepoint 89) and slight increase until the end of the experiment (Timepoint 157) is consistent with in-house titrations of citrate (**Supplementary Figure 4**). -- not bounded; * not detected/data did not exist.
