# Supplementary material for: Continuous in vivo Metabolism by NMR
Source: Front Mol Biosci. 2019 Apr 30;6:26. doi: 10.3389/fmolb.2019.00026 (PMC6502900; doi:10.3389/fmolb.2019.00026)
Supplement: Supplementary file 3 [file Data_Sheet_1.PDF]

Tutorial for CIVM-NMR ridge tracing and quantification

This is the tutorial for the ridge tracing process described in the initial CIVM manuscript. It covers the processes of tracing and quantifying peaks for time series NMR spectra. Before running any code, you need to:

1. Clone the Edison Lab GitHub repository locally. Corresponding functions and workflows can be found at the Edison Lab metabolomics toolbox repository: (https://github.com/artedison/Edison_Lab_Shared_Metabolomics_UGA). Specifically, the pieces of code central to this manuscript are in the folder: metabolomics_toolbox/code/HR_MAS
   In this folder, files with names **STEP*.m** are workflows and other files are functions. This tutorial is mainly for **STEP_2_ridge_tracing.m**, **STEP_3_combining_ridges.m**, and **STEP_4_plotting.m**, and therefore assumes that the sampleData structure has been created using **STEP_1** (only necessary for several samples). Add folders under the GitHub repository to your MATLAB path before running any scripts.

2. Download the necessary supplement data (available at www.metabolomicsworkbench.org). Make sure folders under 'analysis' are in the MATLAB path and ensure MATLAB is running from the 'analysis' folder. The data have been preprocessed in NMRPipe and the MATLAB script (**STEP_1_processing_combine_samples.m**). Processing steps include:
   - line broadening
   - Fast Fourier transform
   - phasing
   - end removal
   - baseline correction
   - solvent region removal
   - normalization

   as stated in the **Methods** part of the paper. Consecutive spectra (every three) have also been summed (collapsed) to improve SNR (signal to noise ratio) and the following working process is quite same for the full resolution dataset. Besides automation of quantification, we also have scripts for automatically constructing project folder (constructHRMASDirectory.m and constructHRMASDirectory_2.m).

Now you can start running the workflow.

1. Ridge tracing

   This part is for the script **STEP_2_ridge_tracing.m**. Go to the script for more technical information if needed.

   1.1. Load data

The data that will be worked on is a struct array containing processed NMR spectra of multiple experiments produced from the workflow (**STEP_1_processing_combine_samples.m**). Specifically, the important part that will be used in the workflow is ppm vector (ppmR_1h1d), collapsed intensity matrix (Xcollapsed_1h1d), and collapsed time vector (timesCollapsed_1h1d). The Xcollapsed_1h1d is a matrix containing time series NMR measurement with each row for different time points and each column for different ppm.

1.2. Smoothing and Peak picking

This step will peak-pick points which can be connected in next step. Based on the specific peak that the user needs to quantify, smoothing and peak density need to be controlled by parameters in this section.

Start from this step, the region of interest (ROI) needs to be specified. The example region is [2.5 2.8] ppm, and regions with similar size should be fine in computational time. Try not to use too large or too small of a region. The former will be slow, and the latter might cause problems with peak picking.

For a selected region, the time series NMR data can be visualized as a 3D surface. On the surface, a 2D Gaussian filter ('imgaussfilt' in MATLAB) is used to smooth the surface. This process can be controlled by setting different sigmas for the dimensions of time and ppm. The value of the sigmas can be increased for noisy regions and decreased if the peaks are close to each other and greater resolution is needed. peakPickThreshold is the threshold for peak picking and it can be decreased to pick more peaks or increased to limit noise (some noise is okay). These parameters are fed to the function ridgeTracing_PeakPick1D for this step. In the example, we are dealing with peaks that are quite intense, so these numbers might need to be modified for smaller peaks. An example figure (Figure 1) produced from this step is shown here with red dots indicating the picked peaks for citrate:

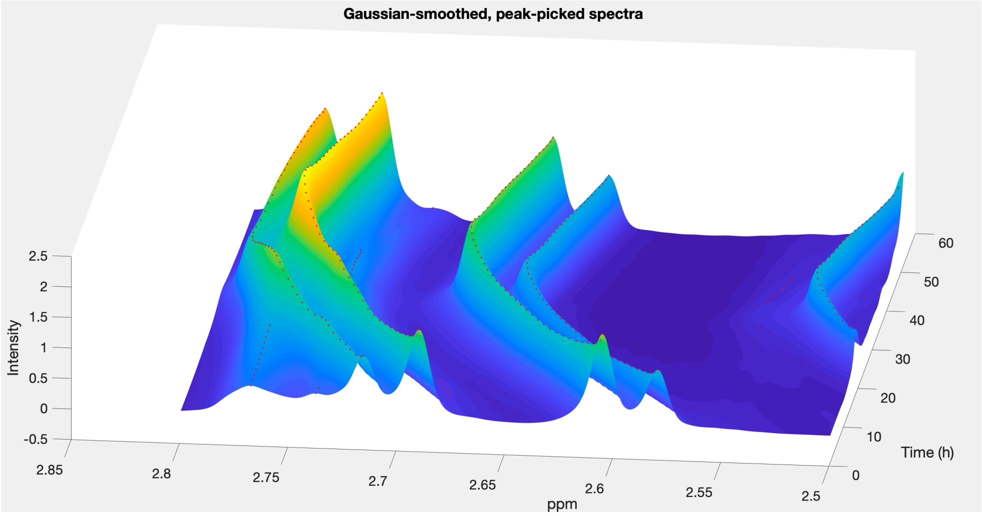

Figure 1: the peak picking result for region [2.5,2.8] with peakPickThreshold 0, sigma in gaussian filter 1 and 10.

Even though step 1.2 and step 1.3 are separated, users will often need to modify parameters on both parts to have a satisfactory result in ridge tracing.

1.3. Clustering and manual picking

This step will cluster the peaks found in last step using single linkage hierarchical agglomerative clustering. The clustering is based on distances in the 3D space of time, intensity, and ppm. For clustering, number of clusters (numberOfRidges), weight in each direction (timeWeight, ppmWeight, and intensityWeight) need to be adjusted for different conditions. For the selected region here, as the peak is shifting, a comparably larger ppmWeight is needed. If you are working on peak that are not moving a lot and comparably crowded, then a smaller ppmWeight is needed. In general, for any dimension, increasing the weight encourages clusters to spread in that corresponding direction. Finally, the number of clusters should typically be set higher than the desired number of peaks to be traced, to allow noise to cluster. The resulting clusters are displayed as 'ridges' on a 3D surface plot. You will often notice noise clustering with noise.

Therefore, manual picking is also needed to select ridges with good quality. By running the function ridgeTracing_clusterPeaks_interactive_2, the peaks are clustered to different ridges with different colors. There will be a menu, from which the user should click Pick Final Clusters, after which the mouse is used to click the ridges to be kept, and then the return key is pressed. The surface plot with high-quality, traced ridges is then displayed (Figure 2). A struct array newRidges will also be returned which contains information for generating the selected ridges and parameters.

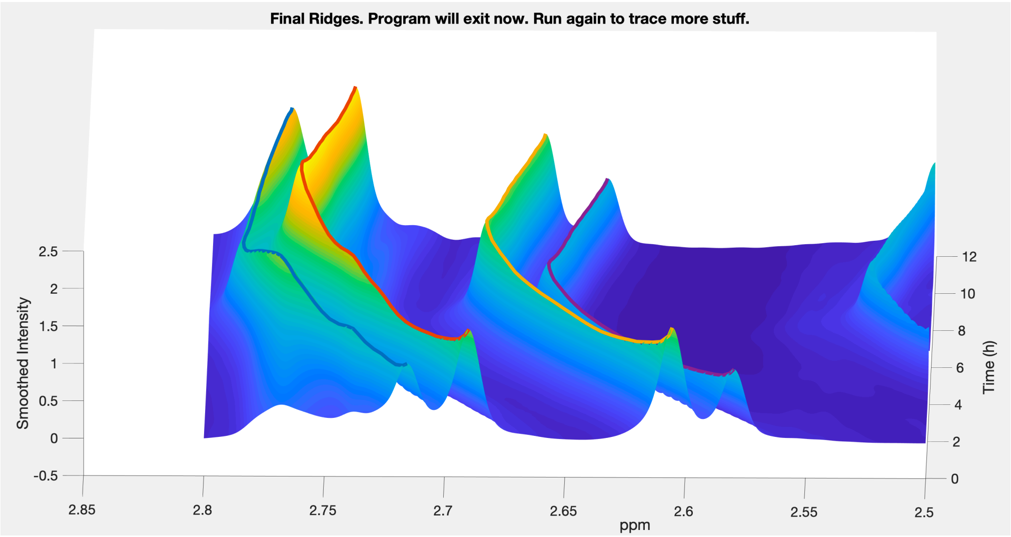

Figure 2: ridges after manual picking with numberOfRidges 13, timeWeight 1, ppmWeight 2, and intensityWeight 500.

1.4. Ridge correction

This step will refine the ridge tracing result. It will map ridges to the original (non-smoothed) matrix without smoothing at the beginning, use linear interpolation to fill gaps, and extend the ridge ends to fill the time series (in case this is required; it typically is not used). A struct array adjustedRidges will be returned containing the refined data. Figures presenting the refined result will also be shown for each ridge. Two parameters, windowWidth and viewWidth need adjustment for this step. Mapping from smoothed data to raw data is done by applying the ridge positions from the former to the data in the latter. Since the true position of a ridge is usually slightly different between the two, the maximum within the region defined by windowWidth data points to the left and right of the smoothed ridge position is taken, and its position and value become the adjusted ridge. viewWidth simply defines the width of the region plotted for inspection.

2. Combing ridge tracing information

From the data of step 2, we can summarize multiple peaks and quantify for each compound. This part is for the script STEP_3_combining_ridges.m. Go to the script for more technical information if needed.

2.1. Adjust time

There is minimal difference in time before the NMR spectral start recording and so for each dataset a time shift is added. Technical details can be found in the script.

2.2. Map ridge to compound and quantification

Quantified ridges can be annotated to specific compound by a direct mapping list. Mapping process depends on the compound annotation on NMR spectra based on extracted sample and chemical annotation experience is needed.

When multiple ridges are annotated to one compound, they are scaled such that the mean of the ridges are the same, then averaged by timepoint. This method of combining ridges facilitates comparison of trends but is not appropriate for absolute quantification.

3. Plotting

Most figures in the manuscript can be produced by the script STEP_4_plotting.m. The figure types and names are detailed indicated in the script. From the script, you can

plot the compound relative concentration through time (Figure 3). Time trajectories for single ridges and baseline can also be shown. Aside from functions mentioned in the script, you can also use the stackSpectra function to plot time series spectra as in Figure 3 in the main text.

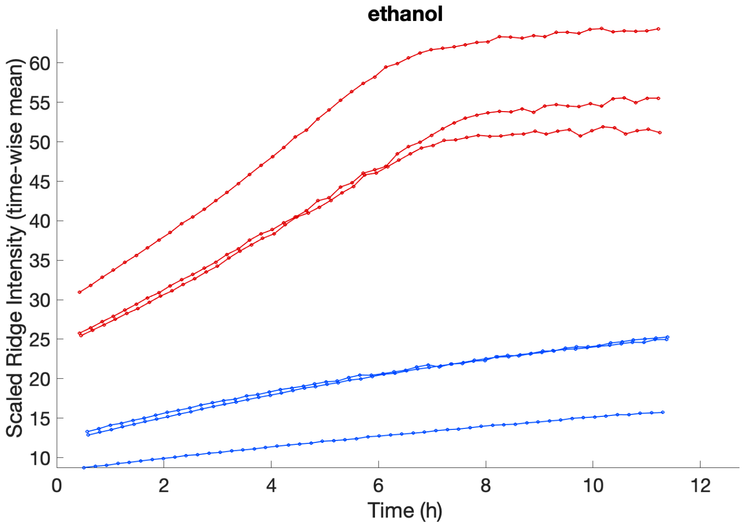

Figure 3: Change of Scaled intensity through time for replicates under different conditions. In this figure two conditions, aerobic and anaerobic are shown and for each case, there are three replicates.

The pipelines and functions for this manuscript are extensively annotated, and if you have more technical questions, please feel free to contact M. Judge or Y. Wu. Additionally, we are working on a more efficient and extensive method for the pipeline which will be described in a later publication, and regularly update the Edison Lab Public Toolbox with new functions and workflows.
