# Supplementary material for: Continuous in vivo Metabolism by NMR
Source: Front Mol Biosci. 2019 Apr 30;6:26. doi: 10.3389/fmolb.2019.00026 (PMC6502900; doi:10.3389/fmolb.2019.00026)
Supplement: Supplementary file 4 [file Image_1.pdf]

Supplementary Material

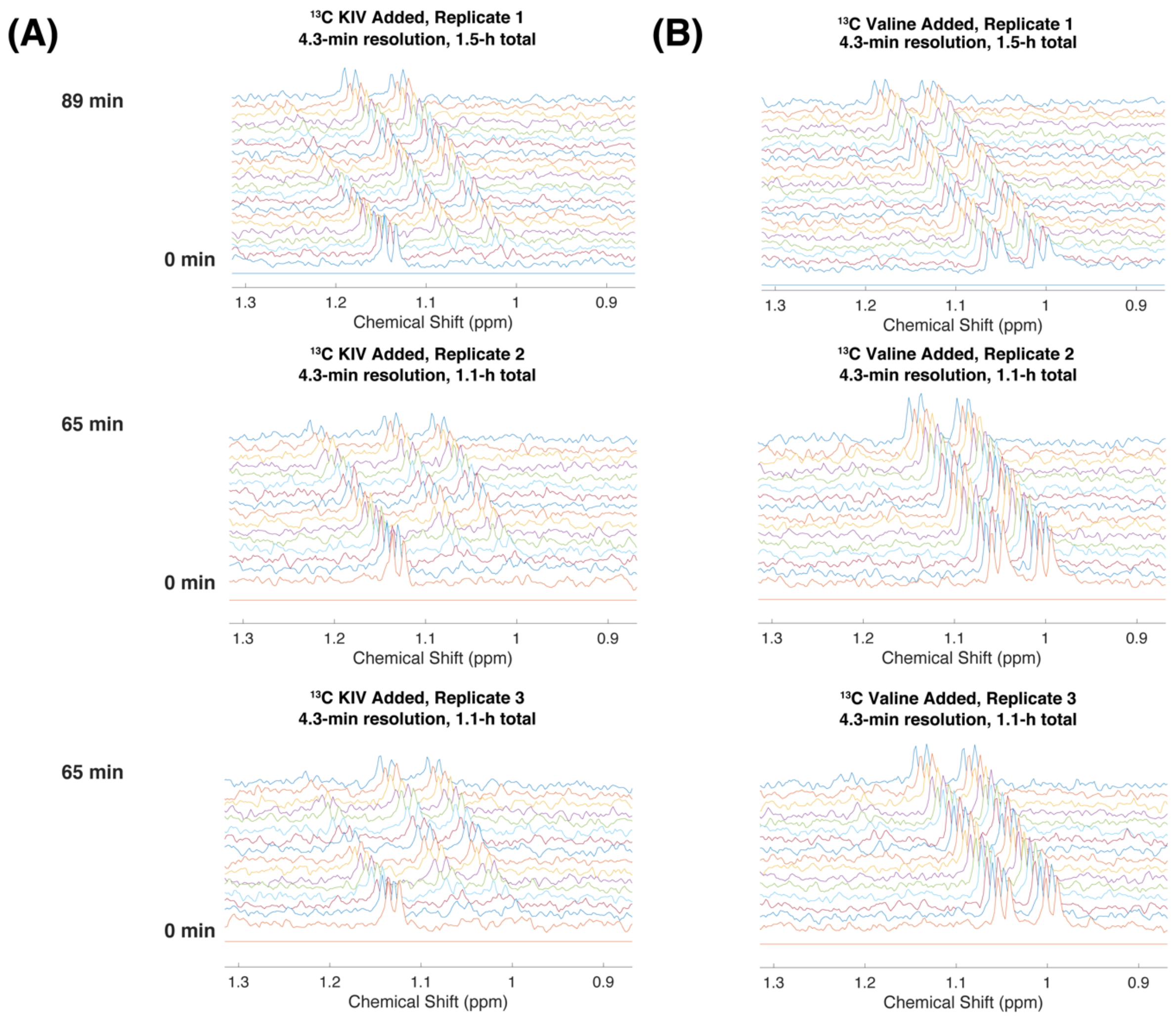

Supplementary Figure 1. Targeted isotopic CIVM-NMR measurements of metabolic flux in human myeloid leukemia cells are reproducible. (A) 1D ^{13}C-edited hsqc experiments were used to observe protons covalently attached to ^{13}C derived from uniformly labeled KIV in three independent replicates. ^{13}C valine accumulates as KIV is consumed. **(B)** The same experiments were used to observe protons covalently attached to ^{13}C derived from uniformly labeled valine in three independent replicates. ^{13}C derived from uniformly labeled valine does not accumulate to appreciable levels in KIV.
