# Supplementary material for: Continuous in vivo Metabolism by NMR
Source: Front Mol Biosci. 2019 Apr 30;6:26. doi: 10.3389/fmolb.2019.00026 (PMC6502900; doi:10.3389/fmolb.2019.00026)
Supplement: Supplementary file 5 [file Image_2.pdf]

Supplementary Material

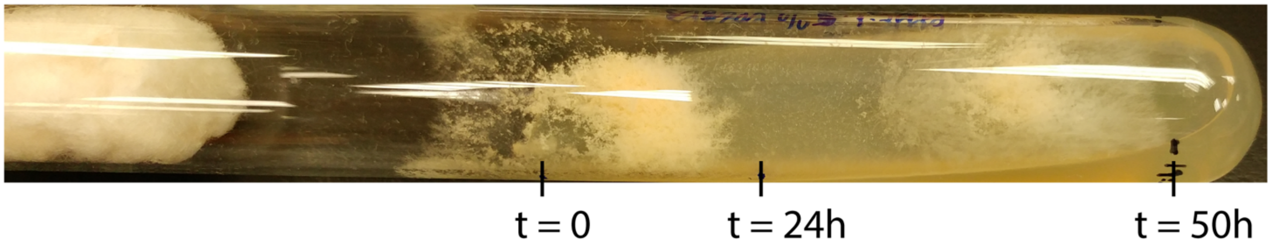

Supplementary Figure 2. Growth of *N. crassa* after a CIVM-NMR experiment. A piece of mycelium was used to inoculate a growth slant at t = 0. The culture was kept on the bench and the advancement of the growth front was marked at t = 0 h, 24 h and 50 h. Roughly circadian conidiation was observed between 0 h and 24 h, and again before the 50-h mark.
