# Supplementary material for: Continuous in vivo Metabolism by NMR
Source: Front Mol Biosci. 2019 Apr 30;6:26. doi: 10.3389/fmolb.2019.00026 (PMC6502900; doi:10.3389/fmolb.2019.00026)
Supplement: Supplementary file 6 [file Image_3.pdf]

Supplementary Material

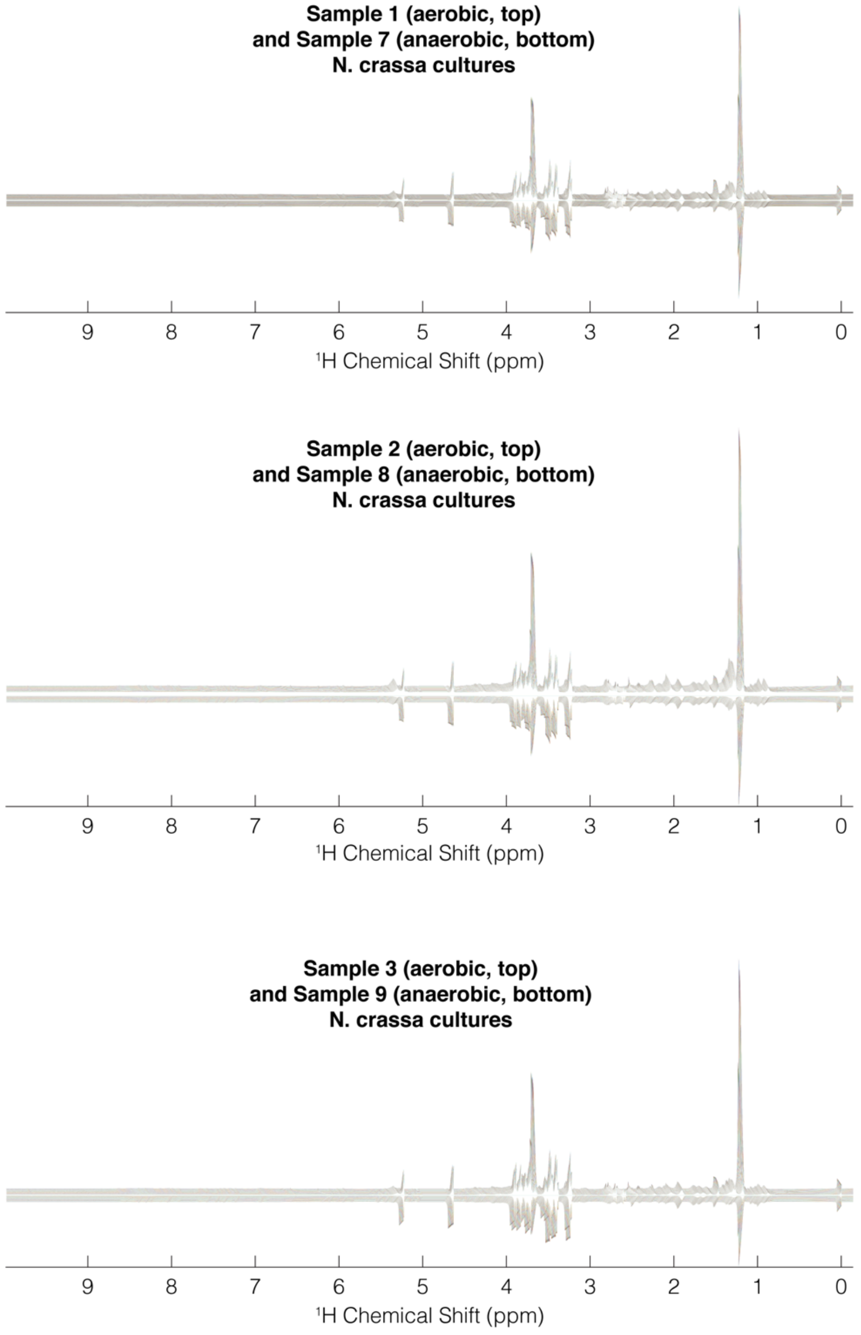

Supplementary Figure 3. Comparison of aerobic and anaerobic samples for three independent replicates. Replicates 1,2,3 (aerobic) are plotted against mirror images of replicates 7,8,9 (anaerobic).
