# Supplementary material for: Continuous in vivo Metabolism by NMR
Source: Front Mol Biosci. 2019 Apr 30;6:26. doi: 10.3389/fmolb.2019.00026 (PMC6502900; doi:10.3389/fmolb.2019.00026)
Supplement: Supplementary file 7 [file Image_4.pdf]

Supplementary Material

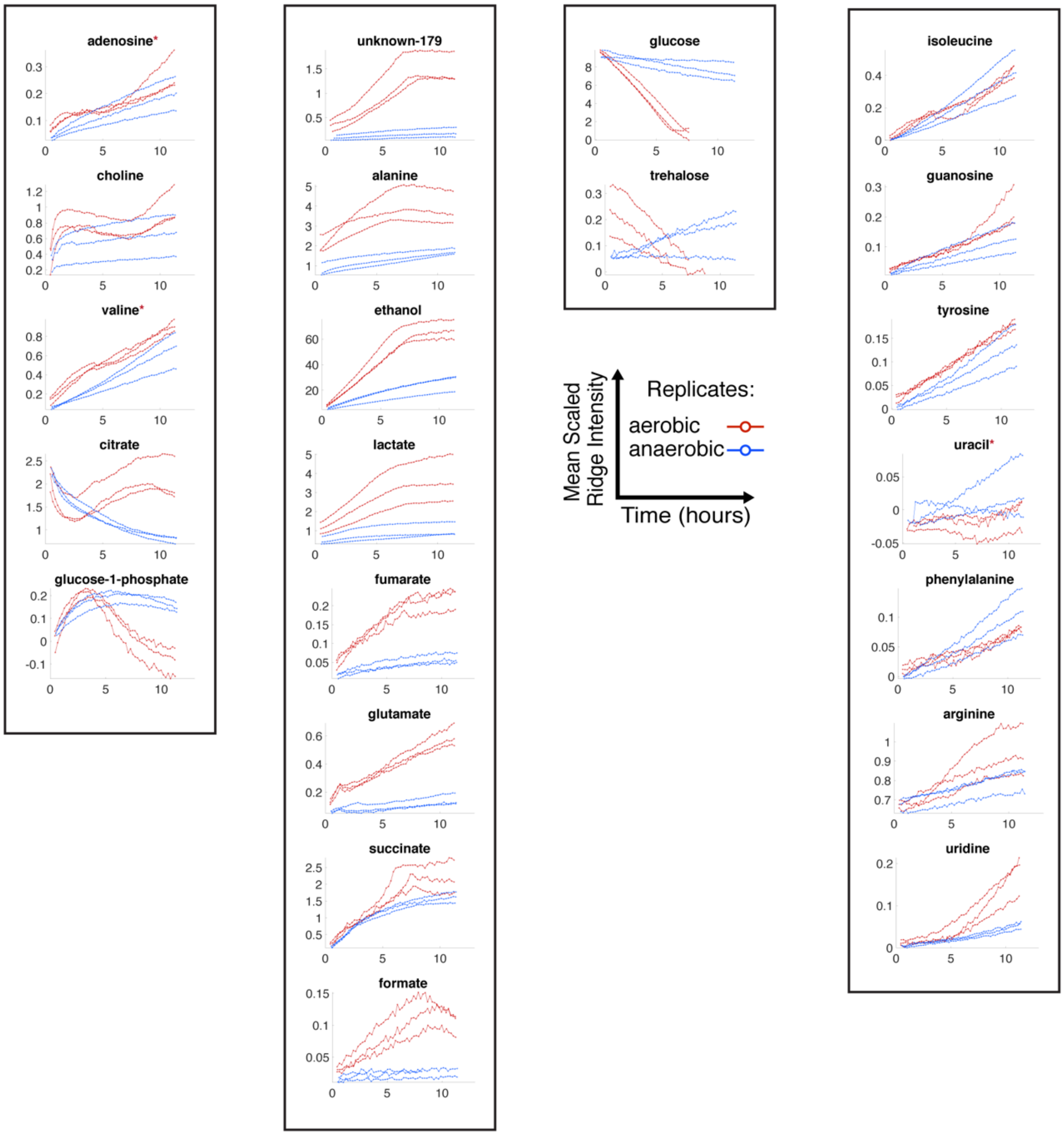

Supplementary Figure 4. Aerobic and anaerobic trajectories for each metabolite that was both annotated and quantified in this study. One example of an un-annotated ridge is also shown. Metabolites are grouped with those having similar profiles in one or both conditions. Red asterisks indicate compounds whose absolute peak intensities were affected by changes in baseline; these were therefore excluded from biological interpretation.
