# Supplementary material for: Continuous in vivo Metabolism by NMR
Source: Front Mol Biosci. 2019 Apr 30;6:26. doi: 10.3389/fmolb.2019.00026 (PMC6502900; doi:10.3389/fmolb.2019.00026)
Supplement: Supplementary file 8 [file Image_5.pdf]

Supplementary Material

(A) **Chemical Shift Changes for Organic Acids in *N. crassa* cultures**

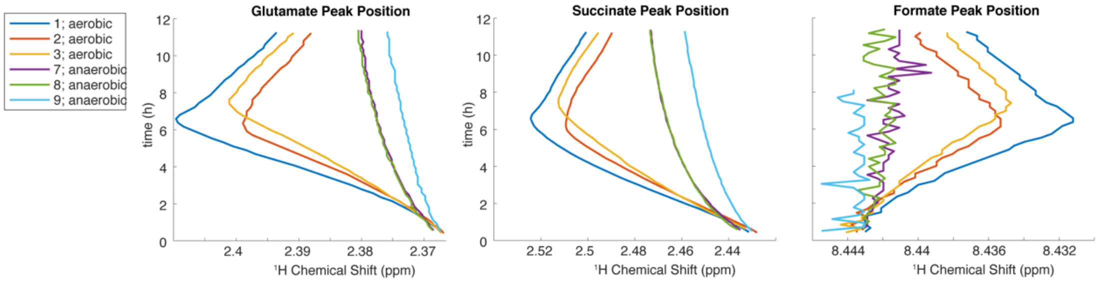

(B) **Citrate Chemical Shift as a Metric for pH of active *N. crassa* cultures**

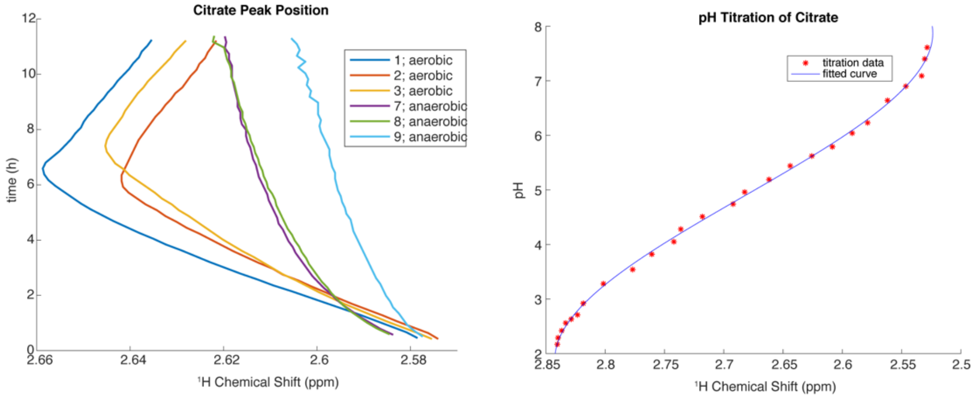

(C) **Modeled pH Changes Correspond with Glucose Consumption and Depletion in *N. crassa* cultures**

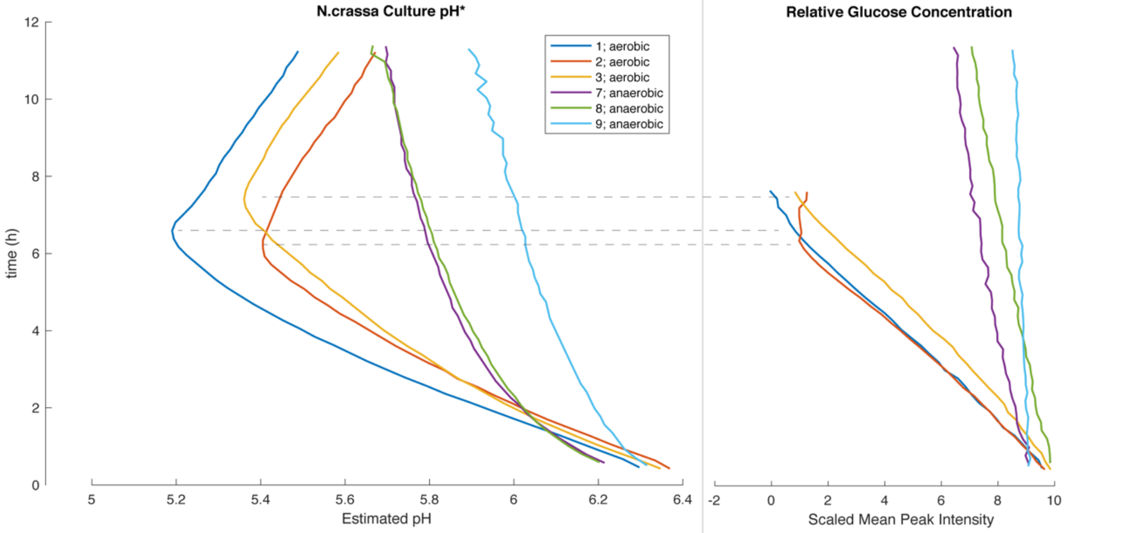

***as determined by citrate peak position**

Supplementary Figure 5. Organic acid peak positions reflect glucose-dependent changes in pH over time. **(A)** Representative *in-vivo* peak position changes for glutamate, succinate, and formate for all samples. The formate peak position in the anaerobic samples is noisy because the peak was very low intensity. **(B)** Position of a representative citrate peak over time and in-house pH titration data for citrate. A 3rd-order polynomial was fit to the titration data in order to interpolate pH values at each timepoint for each sample **(C)**. Dashed horizontal lines are used to show that glucose depletion coincided with reversal of acidification around 6-7 h in the aerobic samples.
