# Supplementary material for: Continuous in vivo Metabolism by NMR
Source: Front Mol Biosci. 2019 Apr 30;6:26. doi: 10.3389/fmolb.2019.00026 (PMC6502900; doi:10.3389/fmolb.2019.00026)
Supplement: Supplementary file 9 [file Image_6.pdf]

Supplementary Material

(A)

Malate Annotated in Endpoint Extract by COLMARm

Peak	database 1H	database 13C	exp 1H	exp 13C
0	2.36	45.32	2.35	45.15
1	2.66	45.32	2.66	45.16
2	4.29	73.06	4.29	72.97

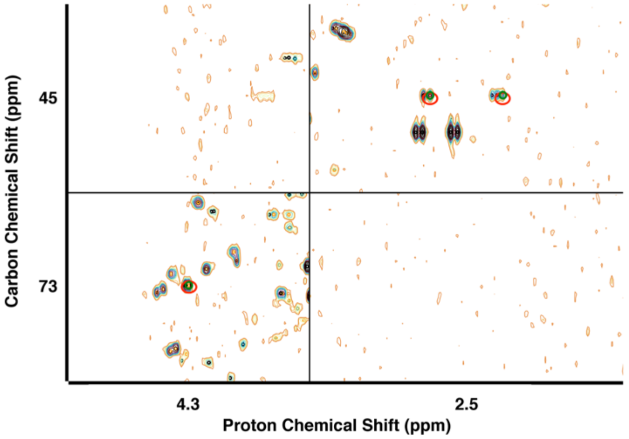

(B)

Putative Glutamine Annotation in *in-vivo* data

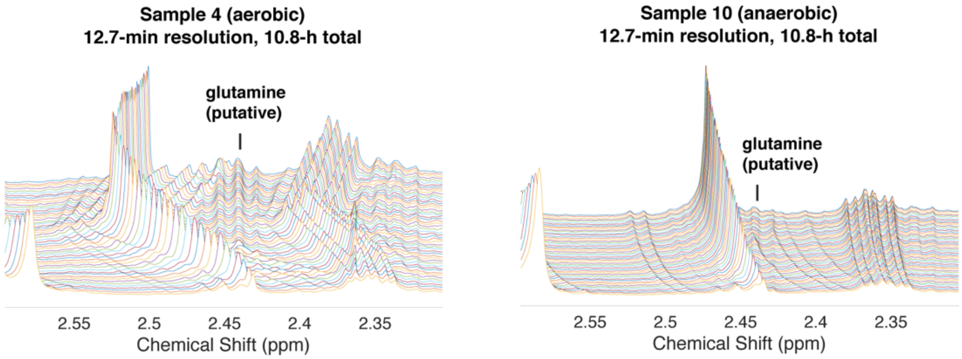

(C)

UDP-GlcNAc and UDP-Glc qualitative changes in *in-vivo* data

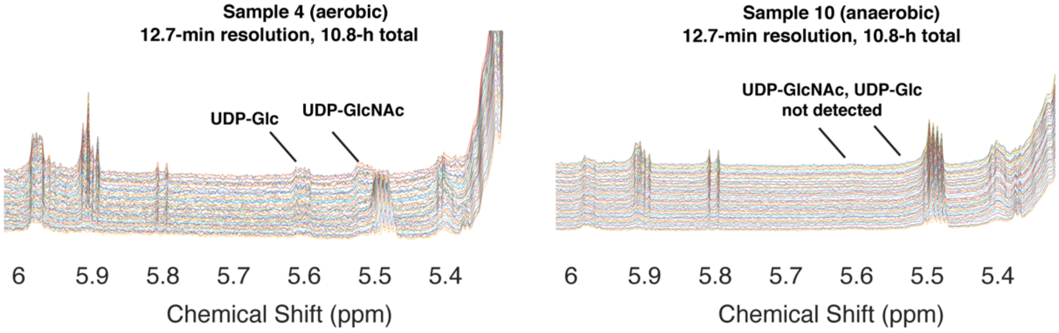

Supplementary Figure 6. Qualitative assessment for unquantifiable metabolites in this study. **(A)** Malate was annotated in the 2D data for endpoint extracts by peak matching to databases using COLMARm[1]. Positions of matched peaks are indicated with a red ellipse and in the peak table. **(B)** Peaks consistent with glutamine increased in the aerobic and may be present in the anaerobic condition. **(C)** UDP-GlcNAc (UDP-N-Acetyl Glucosamine; UDP-NAG) and UDP-Glc (UDP-Glucose) were annotated in the aerobic condition but could not be traced reliably.
