# Supplementary material for: Continuous in vivo Metabolism by NMR
Source: Front Mol Biosci. 2019 Apr 30;6:26. doi: 10.3389/fmolb.2019.00026 (PMC6502900; doi:10.3389/fmolb.2019.00026)
Supplement: Supplementary file 10 [file Image_7.pdf]

Supplementary Material

^{13}C Glucose, 83mM, Aerobic, Replicate 1

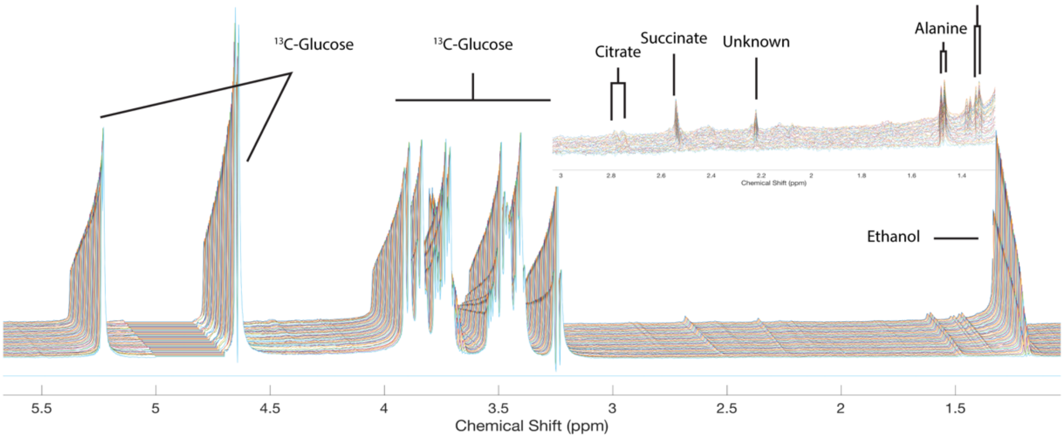

^{13}C Glucose, 83mM, Aerobic, Replicate 2

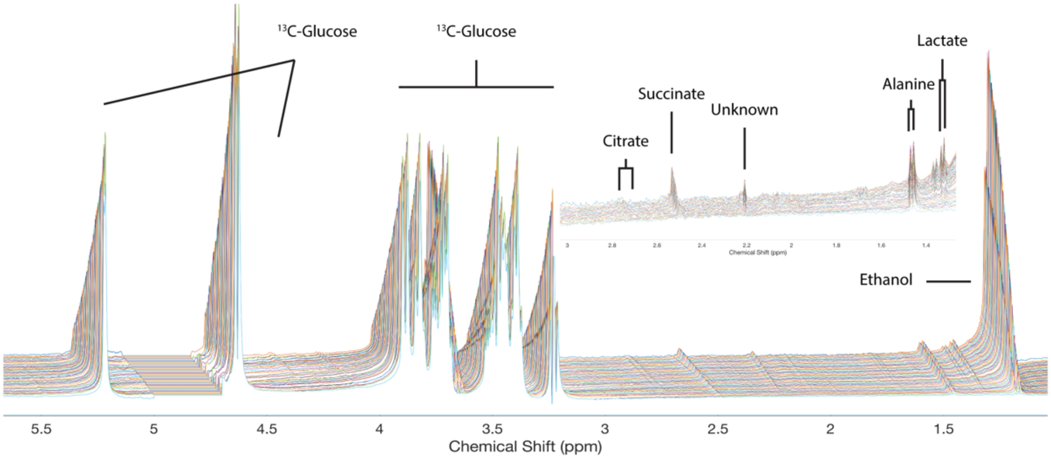

^{13}C Glucose, 83mM, Aerobic, Replicate 3

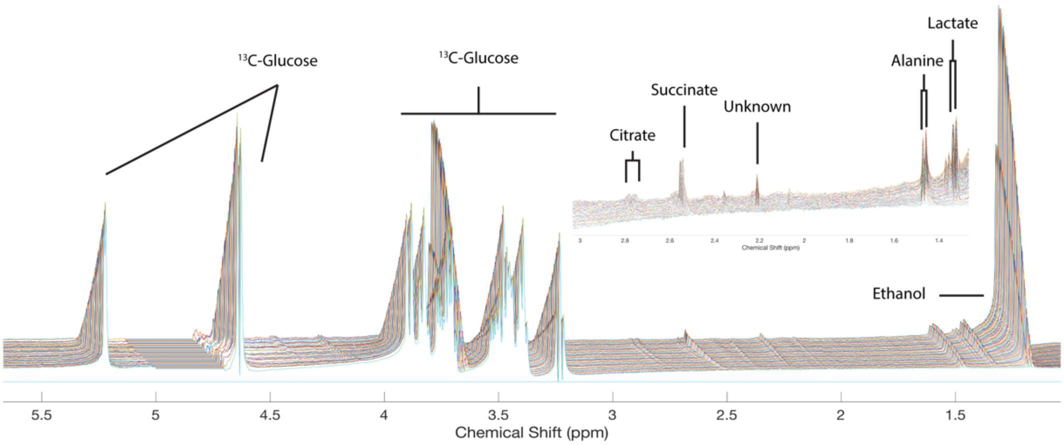

Supplementary Figure 7. Accumulation of ^{13}C-labeled metabolites in three independent replicate aerobic *N. crassa* cultures. hmqc1d NMR experiments were used to monitor the accumulation of ^{13}C-labeled metabolites after addition of uniformly labeled ^{13}C glucose. Glucose was converted to ethanol, alanine, succinate, and lactate, citrate, and some peaks that have yet to be identified.
