# Supplementary material for: Continuous in vivo Metabolism by NMR
Source: Front Mol Biosci. 2019 Apr 30;6:26. doi: 10.3389/fmolb.2019.00026 (PMC6502900; doi:10.3389/fmolb.2019.00026)
Supplement: Supplementary file 11 [file Image_8.pdf]

Supplementary Material

(A)

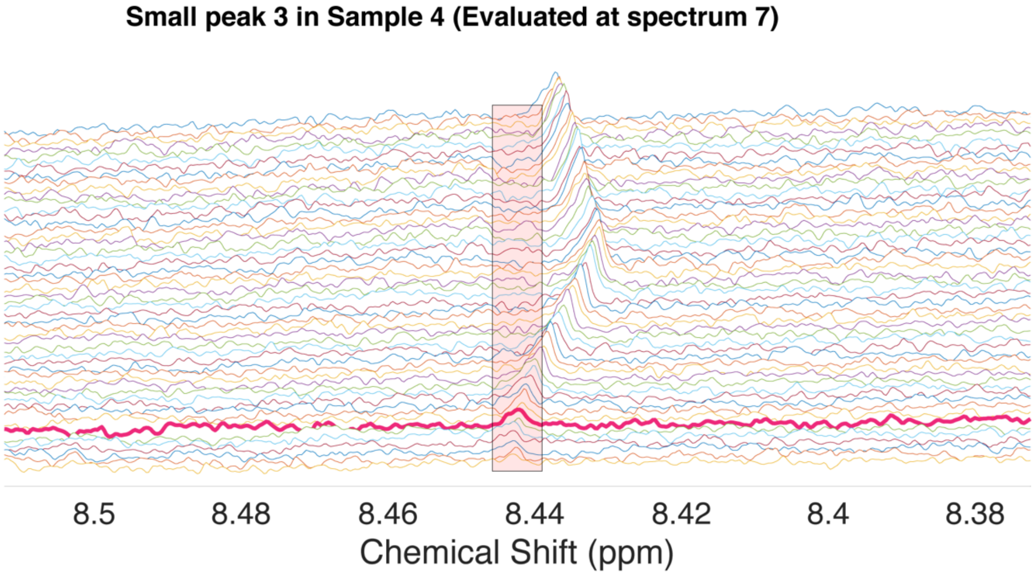

(B)

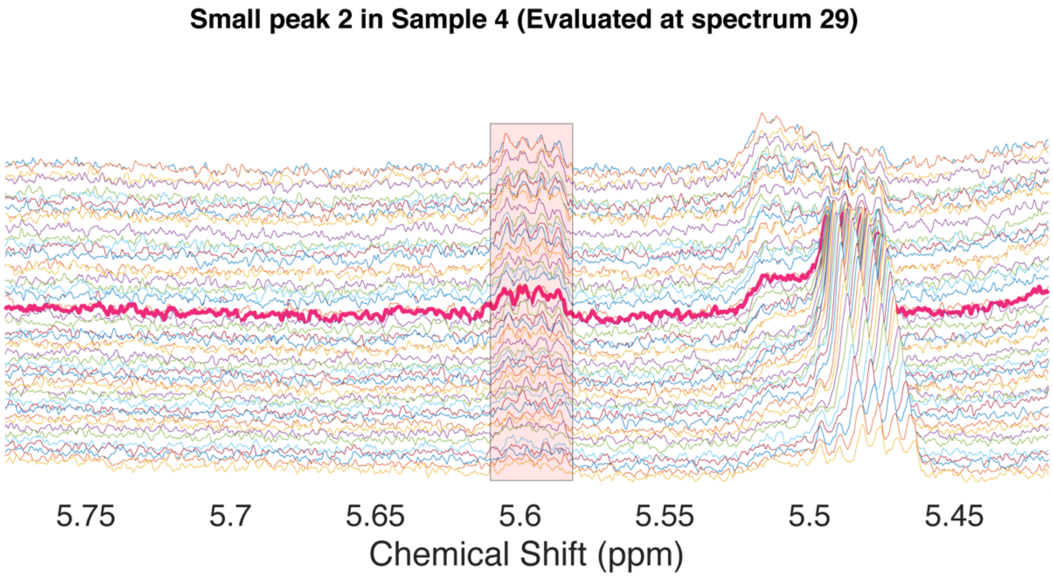

(C)

	Small Peak	Annotation	Integral (Peak)	Integral (DSS)	[1H] (mM)*
Individual Spectra	1	unknown	00.212	0047.526	0.036
	2	UDP-NAG (whole multiplet)	01.474	0048.040	0.246
	2	UDP-NAG (quarter multiplet)	-	-	0.062
	3	formate	00.145	0048.350	0.024
Summed Spectra	1	unknown	20.910	2466.623	0.068
	2	UDP-NAG	69.245	2466.623	0.225

***** [1H] (mM) = (Peak Integral) / (DSS Peak Integral) * (0.89 mM * 9 DSS protons)

Supplementary Figure 8. Estimation of sensitivity for CIVM-NMR using our HR-MAS probe in an aerobic sample. **(A)** Formate peak in spectrum 7 (bolded in magenta, t = 84.6 min), where it became discernable from noise. **(B)** Similar result for a UDP-N-acetyl glucosamine peak in spectrum 29 (t = 363.8 min). **(C)** Table of measured intensities and calculated concentrations of ^{1}H based on the known concentration of DSS in the sample. Integrals were assessed using the boundaries shown by the pink box. Notably, formate and an unknown peak) of similar intensity (not shown) yielded similar concentrations of ^{1}H, and these values are within a factor of ~2 from the calculated potential sensitivity from one quarter of the UDP-N-acetylglucosamine peak.
